# TGFβ promotes YAP‐dependent *AXL* induction in mesenchymal‐type lung cancer cells

**DOI:** 10.1002/1878-0261.12857

**Published:** 2020-12-05

**Authors:** Jeong‐Yun Choi, Haeseung Lee, Eun‐Ji Kwon, Hyeon‐Joon Kong, Ok‐Seon Kwon, Hyuk‐Jin Cha

**Affiliations:** ^1^ College of Pharmacy Seoul National University Korea; ^2^ Intellectual Information Team Future Medicine Division Korea Institute of Oriental Medicine Daejeon Korea; ^3^ Stem Cell Research Center Korea Research Institute of Bioscience and Biotechnology Daejeon Korea; ^4^ Research Institute of Pharmaceutical Sciences Seoul National University Korea

**Keywords:** *AXL*, doxorubicin, EMT, resistance, TGFβ‐SMAD4, YAP

## Abstract

The acquisition of chemoresistance remains a major cause of cancer mortality due to the limited accessibility of targeted or immune therapies. However, given that severe alterations of molecular features during epithelial‐to‐mesenchymal transition (EMT) lead to acquired chemoresistance, emerging studies have focused on identifying targetable drivers associated with acquired chemoresistance. Particularly, *AXL,* a key receptor tyrosine kinase that confers resistance against targets and chemotherapeutics, is highly expressed in mesenchymal cancer cells. However, the underlying mechanism of *AXL* induction in mesenchymal cancer cells is poorly understood. Our study revealed that the YAP signature, which was highly enriched in mesenchymal‐type lung cancer, was closely correlated to *AXL* expression in 181 lung cancer cell lines. Moreover, using isogenic lung cancer cell pairs, we also found that doxorubicin treatment induced YAP nuclear translocation in mesenchymal‐type lung cancer cells to induce *AXL* expression. Additionally, the concurrent activation of TGFβ signaling coordinated YAP‐dependent *AXL* expression through SMAD4. These data suggest that crosstalk between YAP and the TGFβ/SMAD axis upon treatment with chemotherapeutics might be a promising target to improve chemosensitivity in mesenchymal‐type lung cancer.

## Graphical abstract

Through integration of multiple large database, two oncogenic signatures (YAP conserved and TGFβ‐up signatures) were commonly upregulated in the cancer cell lines with the mesenchymal properties. AXL, the expression of which is highly induced in EMT, contributes the doxorubicin resistance in EMT. Doxorubicin treatment induced YAP‐dependent gene response and promoted AXL expression coordinated with TGFβ‐SMAD4.

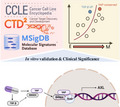

**Abbreviations**
AUC: area under the curve
AXL: AXL receptor tyrosine kinase
BCL2: B‐cell lymphoma 2
CTD2: cancer target discovery and development
CTGF: connective tissue growth factor
DEG: differentially expressed genes
DOXO: doxorubicin
EMT: epithelial–mesenchymal transition
Eto: etoposide
FDA: Food and Drug Administration
ITGB3: integrin beta‐3
MAPK: mitogen‐activated protein kinase
MMP2: matrix metalloproteinase‐2
MMP9: matrix metalloproteinase‐9
mRNA: messenger RNA
NF‐κB: nuclear factor kappa‐light‐chain‐enhancer of activated B cells
SBE: SMAD binding element
SERPINE1: serpin family E member 1
siRNA: small interfering RNA
ssGSEA: single‐sample gene set enrichment analysis
TCGA: The Cancer Genome Atlas
TGFβ: transforming growth factor beta
YAP: Yes‐associated protein
YAP8SA: mutants of inhibitory phosphorylation site at eight serine to Alanine of YAP
ZEB1: zinc finger E‐box binding homeobox 1
ZEB2: zinc finger E‐box‐binding homeobox 2

## 1 Introduction

Despite recent remarkable advances in cancer therapeutics, conventional chemotherapy remains the standard anti‐cancer therapy for lung cancer patients who are not eligible for the most current target therapy or immunotherapies. Therapy resistance acquired during conventional chemotherapy is a major cause of cancer‐related death, and therefore understanding the molecular mechanisms underlying acquired chemoresistance is critical to identify a novel molecular target for chemosensitization [1]. Emerging evidence suggests that molecular alterations during epithelial–mesenchymal transition (EMT) may confer therapy resistance, which results in tumor relapse and mortality [2]. This is consistent with reports that cancer patients with mesenchymal gene signatures exhibit poor prognoses [3].

Therefore, considerable efforts have been made to identify molecular targets associated with EMT‐mediated chemoresistance. Moreover, instead of targeting key transcription factors that govern the EMT process such as ZEB1, SNAIL, SLUG or TWIST, which are poorly druggable, there is a growing interest in identifying druggable targets or developing strategies to ‘drug undruggable targets’ [4, 5, 6] to inhibit EMT‐mediated therapy resistance. For instance, a pharmacological inhibitor of BCL2, a well‐characterized pro‐survival protein whose expression is positively correlated to mesenchymal gene signatures, has been found to inhibit chemoresistance in EMT [7]. Alternatively, *ITGB3* induction driven by the activation of the MAPK pathway leads not only to metastatic potential [8] but also to chemoradioresistance via the NF‐κB survival pathway [9]. Moreover, atorvastatin, an Federal Drug Administration (FDA)‐approved drug, was identified *in silico* to reverse ITGB3‐dependent NF‐κB survival gene response, as similar as *ITGB3* depletion to abrogate the ITGB3‐dependent chemoresistance [10].

The Hippo pathway, which plays pivotal roles in organ development, cell proliferation, survival and homeostasis through the control of YAP/TAZ phosphorylation and nuclear translocation [11], is often deregulated in many types of cancers [12]. The subsequent increase in the transactivation activity of YAP/TAZ through binding to TEA domain transcription factors (TEAD, i.e, highly conserved transcriptional factors in the Hippo pathway, is thought to be a key mechanism for resistance of target therapy [13], as well as conventional chemotherapeutics [14, 15]. Therefore, pharmacological approaches to reactivate the Hippo signaling pathway or interfere with the transactivation activity of YAP/TAZ have been suggested as promising therapeutic alternatives [16, 17]. Moreover, instead of searching FDA‐approved drugs to inhibit YAP‐TEAD indirectly [18], chemical inhibitors to inhibit protein interactions directly between YAP and TEAD have been developed [19, 20].

On the other hand, identifying druggable targets in the downstream effectors of YAP‐TEAD responsible for chemoresistance is another feasible alternative. *AXL* receptor tyrosine kinase (*AXL*), one of the downstream targets of YAP‐TEAD [21], is readily druggable with tyrosine kinase inhibitors [22] and triggers diverse mitogenic and survival signals. Moreover, *AXL* also plays key roles in metastasis, invasion and cancer proliferation [22, 23], as well as EMT [24]. Additionally, *AXL* bypasses the anti‐mitogenic effect of several tyrosine kinase inhibitors (TKI), leading to resistance to target therapeutics [25, 26, 27], and also confers resistance against conventional chemotherapies by promoting survival signals. Thus, *AXL* inhibition can improve the chemotherapy response [28, 29, 30]. Despite recent efforts to demonstrate the roles of *AXL* in typical EMT features (e.g. promoted metastasis and therapy resistance) [24], the molecular mechanisms of *AXL* induction during EMT have not been fully characterized.

In this study, we took advantage of the lung cancer cell model with clear mesenchymal‐like phenotype acquired via EMT (mesenchymal‐type lung cancer cells) [7, 10] and demonstrated that high‐*AXL* expression in mesenchymal‐type lung cancers, which contributes to doxorubicin resistance, was induced by high YAP/TAZ activity upon doxorubicin treatment. Particularly, doxorubicin treatment in mesenchymal‐type lung cancer cells also activated TGFβ‐SMAD4 signaling, which in turn primed YAP/TAZ activity toward *AXL* expression. This priming effect of the TGFβ‐SMAD4 axis toward YAP/TAZ‐dependent *AXL* expression was also validated via a meta‐analysis of lung cancer patient clinical data.

## 2 Materials and methods

### 2.1 Reagents

The primary antibodies against α‐tubulin (#G1417), β‐actin (#sc‐47778), *AXL* (#sc‐166269), PARP (#J2215), YAP (#sc‐101199) were purchased from Santa Cruz Biotechnology (Santa Cruz, CA, USA). Antibodies against cleaved Caspase 3 (#9661), E‐cadherin (#3195), Flag (#14793), phospho‐YAP (#4911s) and Smad4 (#46535) were purchased from Cell Signaling Technology (Danvers, MA, USA). TGFβ (#cyt‐716) was purchased from PROSPEC (Ness‐Ziona, Israel). SB‐431542 (#HYY‐10431) was purchased from MedChemExpress (Monmouth Junction, NJ, USA). Doxorubicin hydrochloride (#44583) was purchased from MERCK (Burlington, MA, USA). Y‐27632 (#1293823) was purchased from Biogems (Westlake Village, CA, USA). Small interfering (si)RNA targeting Negative Control (#SN‐1003) and the others (listed at Tables S1 and S2) were obtained from Bioneer (Daejeon, Korea).

### 2.2 Cell culture

A549, TD and H1299 cell lines were maintained in Dulbecco’s modified Eagle Medium (DMEM) from Hyclone™ (#SH30243.01; Logan, UT, USA), and H358 cell lines were maintained RPMI‐1640 medium from Sigma‐Aldrich (#R8578; St. Louis, MO, USA). DMEM and RPMI‐1640 media were supplemented with 10% (v/v) fetal bovine serum, gentamicin (50 µg·mL^−1^). All cell lines were cultured at 37 °C in a humidified atmosphere of 5% CO_2_ in the air.

### 2.3 Cancer cell‐line transcriptome data analysis

RNA sequencing (RNA‐seq) data and drug‐response profiles of 181 lung cancer cell lines were obtained from the Cancer Cell Line Encyclopedia (CCLE) database (https://portals.broadinstitute.org/ccle) and the Cancer Target Discovery and Development (CTD^2^) data portal (https://ocg.cancer.gov/programs/ctd2/data‐portal/), respectively. In the RNA‐seq data, 19 082 protein‐coding genes were used for subsequent analysis. Transcripts per million (TPM) and expected counts were taken as gene expression levels for single‐sample GSEA (ssGSEA) and differential gene expression analyses, respectively. The cell‐line enrichment scores for 189 known oncogenic signatures were measured by conducting ssGSEA on 181 lung cancer cell lines. The oncogenic signatures were obtained from MSigDB C6 (https://www.gsea‐msigdb.org/gsea/msigdb). For GSEA, differential gene expression analysis between groups (e.g. mesenchymal vs epithelial, YAP‐high vs YAP‐low) was performed using the R package ‘DESeq2’, yielding a ranked list of genes. Using the gene list as the input, GSEA was run via the R package ‘fgsea’ for public annotated gene sets of interest (e.g. KEGG pathways, oncogenic signatures).

### 2.4 The Cancer Genome Atlas (TCGA) transcriptome data analysis

RNA‐seq and clinical data of lung adenocarcinoma patients in the TCGA LUAD cohort were obtained from the Broad GDAC firehose (https://gdac.broadinstitute.org/). Overall, 515 patients with clinical information tracked over at least 1 month were used. For survival analysis, patients were divided into three groups according to tertiles of the expression level of the gene of interest (e.g. *AXL* or YAP1) or mesenchymal score. TPM was taken as gene expression levels. Upper and lower groups excluding the intermediate were defined as ‘high’ and ‘low’ groups. Recurrence‐free survival analysis was performed to test the difference in the recurrence‐free survival rate between the groups by using the R package ‘survival’. The hazard ratio (HR) and *P*‐value (*P*) were computed using Cox proportional hazards regression analysis and the log‐rank test, respectively. Differential gene expression analysis between *AXL*‐high and *AXL*‐low groups was performed and genes that met the criteria of |log_2_ fold change | ≥ log_2_3 and FDR ≤ 10^−3^ were selected as differentially expressed genes (DEG).

### 2.5 Transfection (plasmid DNA and siRNA)

Transient transfection of plasmid DNA or siRNA was performed with Lipofectamine 2000 (Invitrogen, Waltham, MA, USA) following the manufacturer’s protocol. The DNA construct used is described as follows: 8X GTIIC‐luciferase, Myc‐TEAD4, Flag‐YAP wild type (WT) and Flag‐YAP 8SA were kindly gifted by M. Jung‐Soon (Ajou University, Suwon, Republic of Korea).

### 2.6 RNA extraction and quantitative real‐time PCR

Total RNA was extracted using easy‐BLUE™ Total RNA Extraction Kit (#17061; iNtRON, Seongnam, Korea) followed by RT‐PCR to generate the cDNA (cat#RR036A; Takara, Shiga, Japan), and the cDNA was applied to real‐time PCR with TB‐Green (cat#RR420; Takara) following the manufacturer’s protocol. Sequence information for RT‐PCR primer and siRNA information is as follows.

**Table:** 

**Gene symbol**	**Primer sequence (5′ to 3′)**
GAPDH	F: GCA TCC TGC ACC ACC AAC TG R: GCC TGC TTC ACC ACC TTC TT
AXL	F: GTG GGC AAC CCA GGG AAT ATC R: GTA CTG TCC CGT GTC GGA AAG
ANKRD1	F: AGT AGA GGA ACT GGT CAC TGG R: TGG GCT AGA AGT GTC TTC AGA T
AREG	F: GCC GCT GCG AAG GAC CAA TG R: CCA GCA GCA TAA TGG CCT GAG CC
BIRC5	F: GGA CCA CCG CAT CTC TAC R: GCA CTT TCT TCG CAG TTT
CDH1	F: TGC CCA GAA AAT GAA AAA CG R: GTG TAT GTG GCA ATG CGT TC
CDH2	F: GAC AAT GCC CCT CAA GTG TT R: CCA TTA AGC CGA GTG ATG GT
MMP2	F: TAC AGG ATC ATT GGC TAC ACA CC R: GGT CAC AT GCT CCA GAC T
MMP9	F: TGT ACC GCT ATG GTT ACA CTC G R: GGC AGG GAC AGT TGC TTC T
SERPINE1	F: TTG AAT CCC ATA GCT GCT TGA AT R: ACC GCA ACG TGG TTT TCT CA
SMAD4	F: GTC TGG CTT AAG GAG AGC CAT ACT R: GATACCTGCAACTCACCTTCCTAC
TGFB1	F: CAA GTG GAC ATC AAC GGG TTC AC R: GTC CTT GCG GAA GTC AAT GTA CAG
YAP	F: GTG AGC CTG TTT GGA TGA TG R: CAC TGG ACA AAG GAA GCT GA
ZEB1	F: AAG AAT TCA CAG TGG AGA GAA GCC A R: CGT TTC TTG CAG TTT GGG CAT T
ZEB2	F: GAA GAC AGA CAG TGG CAT GTA TGC R: GAG TGC TCG ATA AGG TGG TGC TTG
**Gene Symbol**	**siRNA sequence (5′ to 3′)**
AXL	S: GAC UGU CUG GAU GGA CUG U AS: ACA GUC CAU CCA GAC AGU C
SMAD4	S: GAG ACA UUU AAG GUU CCU U AS: AAG GAA CCU UAA AUG UCU C
TEAD4	S: CCG CCA AAU CUA UGA CAA ATT AS: UUU GUC AUA GAU UUG GCG GTT
YAP	S: CAG AAG AUC AAA GCU ACU U AS: AAG UAG CUU UGA UCU UCU G

### 2.7 Immunoblotting and immunofluorescence

Cell lysates were extracted by RIPA buffer supplemented with 1% protease inhibitor cocktail and 0.1% sodium orthovanadate. After 1 h incubation on ice, total protein was extracted by centrifugation. The concentration of total protein was quantified by BCA protein assay kit (#23225; Thermo Scientific™; Waltham, MA, USA). Approximately 25 µg of total protein was separated on various concentrations of SDS/PAGE (7.5%, 10% or 15%). Separated protein in the gel was transferred to PVDF membrane. Membrane with protein was blocked with 5% skim milk in Tris‐buffered saline with 0.1% Tween‐20 (TBS‐T) for 1 h and then washed three times by TBS‐T for 5 min each. The membrane was incubated with primary antibody in TBS‐T (1 : 1000) with 0.1% sodium azide at 4 °C overnight. Incubated membrane was washed three times with TBS‐T for 5 min each. The membrane was incubated at room temperature with HRP‐conjugated secondary antibody (Jackson ImmunoResearch Laboratories, West Grove, PA, USA) in TBS‐T (1 : 10 000) for 1 h. Incubated membrane was washed three times with TBS‐T for 15 min each. Immunoreactivity was detected by Chemi‐Doc using WEST‐Queen™ (#16026; iNtRON Biotechnology) kit. For immunofluorescence, cells were fixed 4% paraformaldehyde or cold methanol for 10 min, and permeabilized with 0.1% Triton X‐100 in PBS for 3 min (in the case of methanol, the permeabilization step is not necessary). The fixed cells were then incubated overnight at 4 °C in TBS‐T with 3% BSA for blocking. Next, the cells were washed three times in TBS‐T for 15 min each and incubated with primary antibody (1 : 200) in TBS‐ T in a humidity chamber for 1 h. The cells were then washed three times in TBS‐T for 5 min each and incubated with Alexa 594 conjugated secondary antibody or Cy2‐conjugated secondary antibody (1 : 200) and 0.5 mg·mL^−1^ 40, 6‐diamidino‐2‐phenylindole (DAPI) in a dark humidity chamber for 1 h. Finally, the cells were washed in three times TBS‐T for 15 min each in the dark and attached to slide glass using Mowiol solution.

### 2.8 Dual‐luciferase assay

Cells were transfected with specific promoter‐luciferase vector and pRL vector following the above procedures. Cells lysates were extracted with 1× passive lysis buffer. After 1 h incubation on ice, total lysate was extracted by centrifugation. The supernatant was reacted using LARII and Stop & Glo reagent. The reporter assay was performed according to the Dual‐Luciferase Reporter Assay System (#E1980; Promega).

### 2.9 Transwell invasion assay

Transwell (6.5 mm) with an 8‐µm pore polycarbonate membrane insert (from Corning, Corning, NY, USA) was embedded with 120 µg Matrigel (from Biosesang, Seongnam, Gyeonggi, Korea) and 100 µg gelatin (from Sigma‐Aldrich) coats at the bottom of the membrane. Cells were added into the Matrigel‐embedded insert with serum‐free RPMI media, and the inserts were placed into the bottom chambers containing 10% FBS media. Cells were incubated for 24–48 h. The images were taken with a light microscope.

### 2.10 Zymography

Cells were starved for 24 h with DMEM containing 2% FBS. Supernatants were concentrated using a 30‐kDa cut centricon. After adding non‐reducing sample buffer, concentrated proteins were separated on 8% gel containing gelatin by electrophoresis. The gel was washed three times by secondary washing buffer for 30 min each and incubated with reaction buffer at 37 °C overnight. After reaction, the gel was stained with 0.25% Coomassie brilliant blue solution (#C1031; Biosesang) and destained with destaining buffer.

### 2.11 Cell proliferation

JuLI stage (NanoEntek, Seoul, Korea) was used to measure the cell proliferation rate. After cell seeding, plates were loaded on the JuLI stage for 2 days. With the JuLI stage, time‐dependent live images were obtained and then analyzed using JuLI‐Stat software (NanoEntek, Seoul, Korea) to determine time‐dependent growth potential, according to the manufacturer’s manual.

### 2.12 Statistical analysis

Graphical data are presented as mean ± standard deviation (SD). Statistical significance among three groups and between groups was determined using Student’s *t*‐test. Significance was assumed for *P* < 0.05(*), *P* < 0.01(**) and *P* < 0.001(***).

## 3 Results

### 3.1 High YAP activity in mesenchymal‐type lung cancer cells

**Fig. 1:**
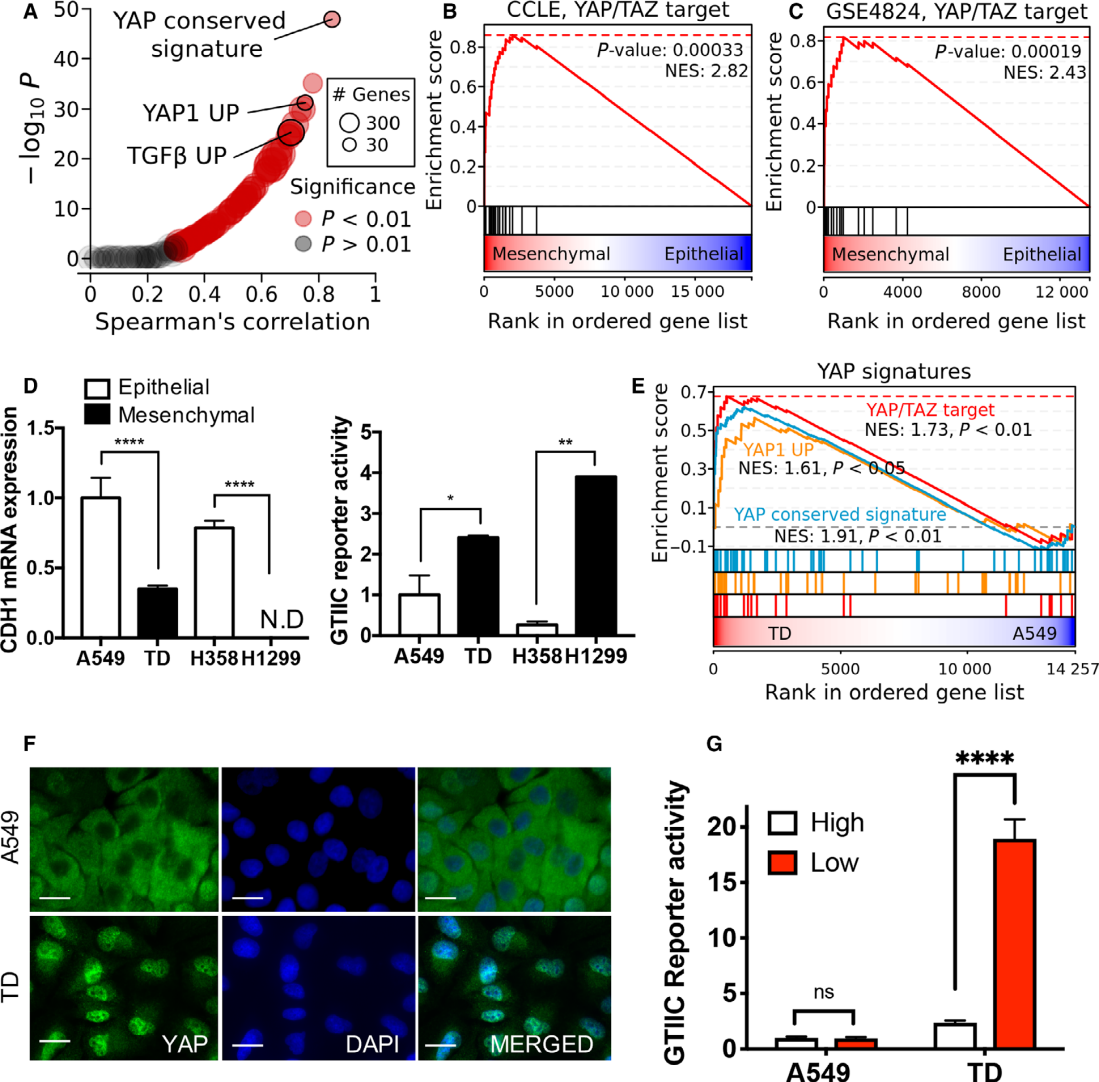
High YAP activity in chemoresistant mesenchymal cancer cell. (A) Correlation between cell‐line mesenchymal score and enrichment score across 189 oncogenic signatures. (B,C) Gene set enrichment analysis (GSEA) plots showing the enrichment of YAP/TAZ target genes in a ranked list of genes differentially expressed between mesenchymal and epithelial cell groups in (B) CCLE and (C) GEO (GSE4824) datasets. (D) *CDH1* mRNA expression level in H358, H1299, A549 and TD cells (left) and reporter activity of GTIIC promoter (right), N.D., not determined. (E) GSEA plot showing the enrichment of three YAP signatures in a ranked list of genes differentially expressed between TD and A549 cells. (F) Immunofluorescence microscopic image for YAP (green) of A549 and TD cells, DAPI (blue) for nuclear staining (600×, scale bar: 50 μm). (G) Reporter assay of GTIIC promoter activity in A549 and TD cells (High: High cell density, Low: Low cell density), Mean ± SD, Student’s *t*‐test, *P* < 0.05(*), *P* < 0.01(**) and *P* < 0.0001 (****), *n* = 3.

To identify dysregulated oncogenic pathways driving lung cancer EMT and therapy resistance, we evaluated the gene expression data of 181 lung cancer cell lines with cell‐line mesenchymal scores calculated as described in a previous study [31]. Using 189 publicly available oncogenic gene sets from MSigDB [32], enrichment scores were computed for 181 lung cancer cell lines via ssGSEA. Correlation analyses between cell‐line oncogenic enrichment scores and mesenchymal scores identified YAP as a key oncogene activated in mesenchymal‐type lung cancer cells, in addition to TGFβ, a well‐characterized EMT inducer (Fig. 1A, Table S1). YAP/TAZ target genes associated with the Hippo signaling pathway [33] were also significantly enriched in mesenchymal state lung cancer cells obtained from the CCLE (Fig. 1B and Fig. S1) or GSE4824 (Fig. 1C and Fig. S1B) transcriptome datasets, which is consistent with previous studies demonstrating the EMT‐promoting activity of YAP [34, 35].

Our previous studies [7, 8, 10, 36] and other authors [37] have previously reported that EMT characteristics such as high invasiveness and chemoresistance in an isogenic lung cancer cell model (A549 transdifferentiated cells: A549TD, hereafter referred to as TD) were established by long‐term exposure to TGFβ. Along with this isogenic pair, we characterized the YAP reporter activity in four in‐house non‐small cell lung carcinoma (NSCLC) lines with epithelial or mesenchymal characteristics (epithelial: H359 and A549, mesenchymal: H1299 and TD). As predicted, H1299 and TD cells with a low expression of *CDH1* (encoding E‐cadherin), a typical epithelial marker (Fig. 1D, left panel), and a high expression of *ZEB2* (Fig. S1C) exhibited relatively high GTIIC reporter activity, a reporter system used as an indicator of YAP/TAZ transcriptional activity [38] (Fig. 1D, right panel). To examine further the positive correlation between YAP activity and EMT properties, we took advantage of the A549 and TD isogenic pair to minimize any cell‐type‐specific bias. Consistent with the upregulation of YAP signatures in TD compared with A549 (Fig. 1E), the nuclear localization of YAP (Fig. 1F and Fig. S1D), a robust indicator of YAP activation, and high reporter activity at low cell density, which inhibits Hippo signaling activity (Fig. 1G), were observed in TD cells. These results demonstrated that YAP activity was elevated in mesenchymal‐type lung cancer cell lines including TD cells.

### 3.2 Identification of *AXL* as a factor in doxorubicin chemoresistance

**Fig. 2:**
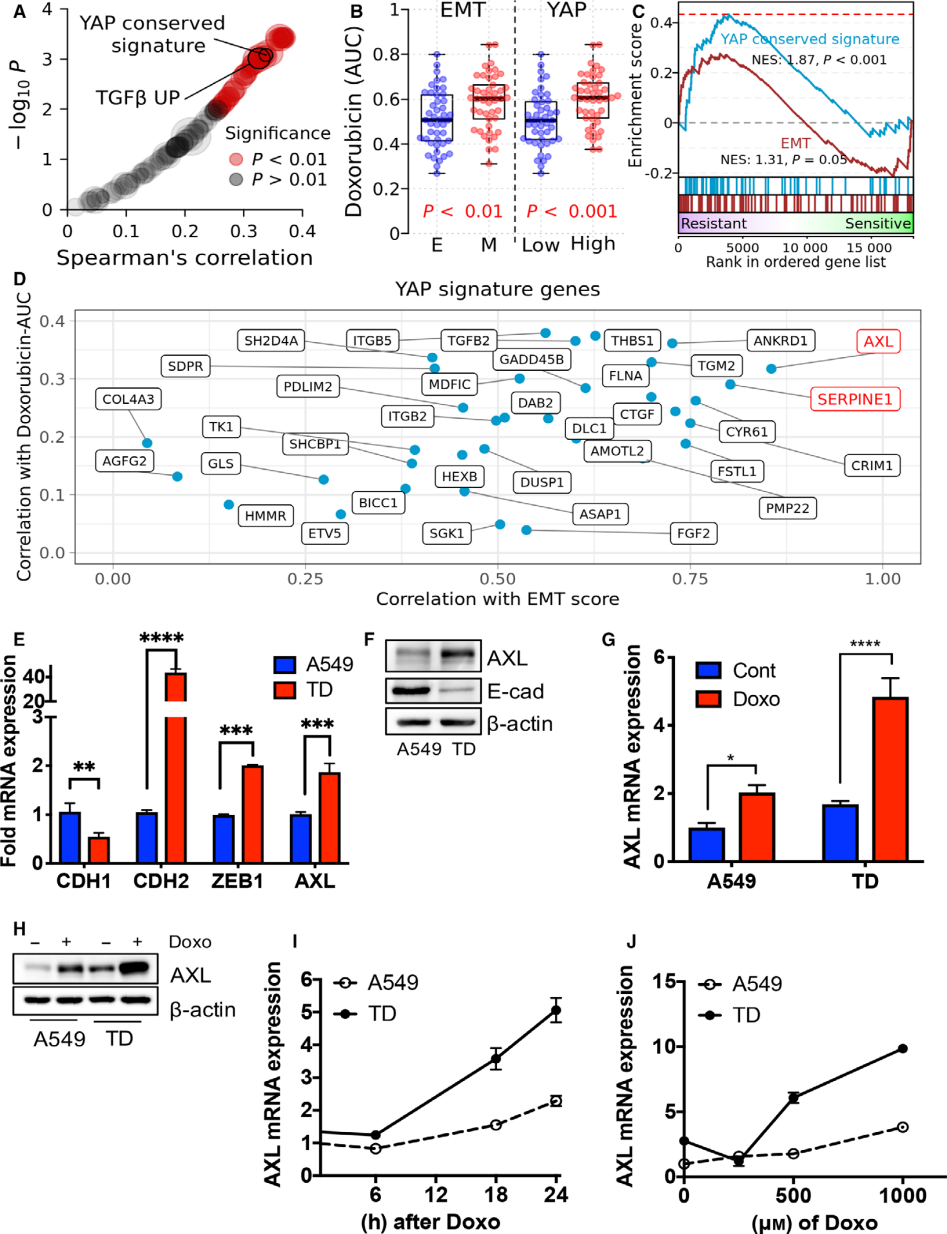
Identification of *AXL* as a player for doxorubicin chemoresistance. (A) Correlation between cell‐line drug sensitivity measurements (AUC) and enrichment score across oncogenic signatures. (B) Distribution of doxorubicin sensitivity by the cell groups of EMT (epithelial: E and mesenchymal: M) and YAP activity (YAP‐low: Low and YAP‐high: High) in lung cancer cell line. *P‐*values were calculated with two‐tailed *t*‐tests. (C) GSEA plot of EMT and YAP signatures in a ranked list of genes differentially expressed between doxorubicin‐resistant and doxorubicin‐sensitive cell groups. (D) YAP signature genes scattered by their expression correlation with mesenchymal score and correlation with sensitivity to doxorubicin. (E) Real‐time PCR analysis of each indicative gene in A549 and TD cells. (F) Immunoblotting analysis for indicative proteins in A549 and TD cells, β‐actin for equal protein loading. (G) *AXL* mRNA expression in A549 and TD cells with or without doxorubicin (Doxo, 1 μm) treatment. (H) Immunoblotting analysis for AXL protein in A549 and TD cells with or without doxorubicin (1 μm) treatment. (I) *AXL* mRNA expression in A549 and TD cells at the indicative time after doxorubicin (1 μm) treatment. (J) *AXL* mRNA expression in A549 and TD cells for each concentration of doxorubicin treatment, Mean ± SD, Student’s *t*‐test, *P* < 0.05(*), *P* < 0.01(**), *P* < 0.001(***) and *P* < 0.0001(****), *n* = 3.

We previously demonstrated that TD cells were highly resistant to doxorubicin [10] and etoposide [7]. To examine whether YAP activity also drives doxorubicin‐specific chemoresistance in lung cancer cells, we compared measurements of cell‐line sensitivity to doxorubicin in the CTD^2^ dataset with the cell‐line oncogenic enrichment scores. Notably, the YAP conserved signature was markedly enriched in the doxorubicin‐resistant cell lines along with the TGFβ signature, which was shown to be highly correlated with the EMT signature via a comprehensive meta‐analysis [39] (Fig. 2A, Table S2). Similar to doxorubicin, other chemotherapeutics such as topotecan and gemcitabine also showed a strong correlation to these signatures across all lung cancer cell lines (Fig. S2A).

According to CTD^2^ data, mesenchymal cell lines and cell lines with high YAP activity had an increased resistance to doxorubicin (Fig. 2B and Fig. S2B). In particular, high YAP activity appeared to be more closely related to lung cancer resistance to doxorubicin compared with the mesenchymal state (Fig. 2C and Fig. S2C). Therefore, we aimed to identify key genes among the YAP signature genes associated with mesenchymal status and lung cancer chemoresistance. *AXL* and *SERPINE1* were selected as candidate genes because their expression was most positively correlated with the cell‐line mesenchymal score and drug resistance to doxorubicin (Fig. 2D). Consistent with this, the role of AXL and PAI‐1 (encoded by *SERPINE1*) in therapy resistance has been previously reported [26].

TD cells that exhibited resistance to doxorubicin [10] and etoposide [7] had a high‐*AXL* expression compared with their parent A549 epithelial cells (Fig. 2E,F). *AXL* was markedly induced upon doxorubicin treatment (Fig. 2G,H) in a dose‐ and time‐dependent manner (Fig. 2I,J). Furthermore, *SERPINE1* was markedly induced in TD cells upon doxorubicin treatment (Fig. S2D). Etoposide, another genotoxic chemotherapeutic, induced *SERPINE1* and *AXL* in TD cells (Fig. S2E). These data suggest that YAP activation (Fig. 1) may induce *AXL* or *SERPINE1* in mesenchymal‐type lung cancer cells upon treatment with genotoxic chemotherapeutic compounds.

### 3.3 YAP activity induces AXL upon doxorubicin treatment

**Fig. 3:**
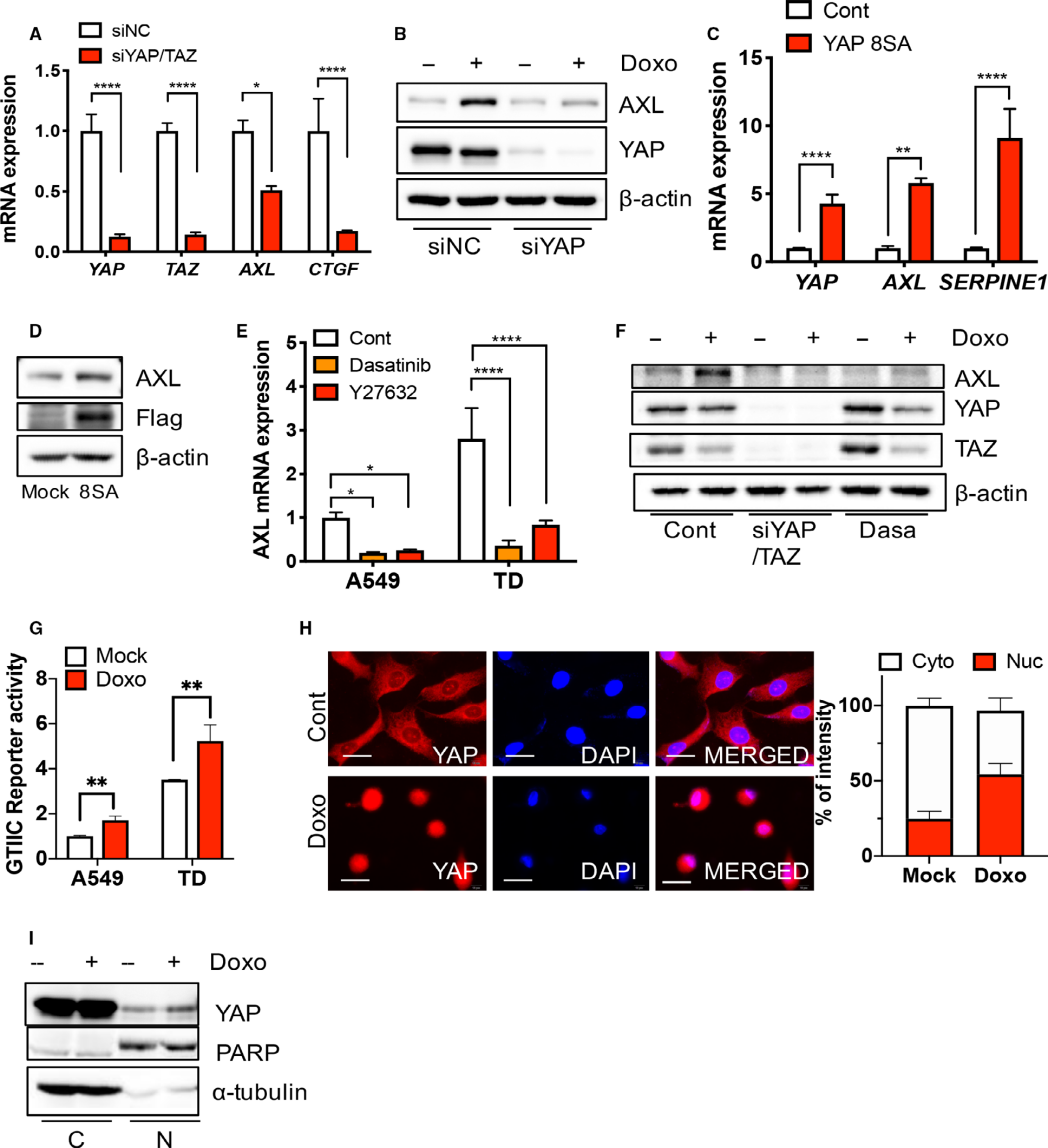
YAP activity for *AXL* induction upon doxorubicin. (A) Real‐time PCR analysis of each indicative gene after introduction of control (siNC) or YAP/TAZ (siYAP/TAZ) siRNA. (B) Immunoblotting analysis for indicative proteins in TD cells after introduction of control (siNC) or YAP (siYAP) siRNA, upon doxorubicin (Doxo, 1 μm) treatment. (C,D) Real‐time PCR analysis (C) and immunoblotting analysis of the indicative gene or protein after introduction of control or YAP8SA in A549 cells. (E) Real‐time PCR analysis of *AXL* expression after Dasatinib (100 μm) and Y27632 (10 ng·mL^−1^) in A549 and TD cells (F) immunoblotting analysis of the indicative proteins with siRNA for YAP/TAZ (siYAP/TAZ) or 100 μm Dasatinib (Dasa), 24 h after 1 μm doxorubicin treatment (Doxo) in TD cells. (G) Reporter assay of GTIIC promoter activity in A549 and TD cells after doxorubicin (Doxo, 1 μm) treatment. (H) Immunofluorescence microscopic images for YAP (red) in TD cells, DAPI (blue) for nuclear staining (left). Graphical presentation of cytoplasmic (Cyto) and nuclear intensity (Nuc) of YAP in A549 cells after doxorubicin (Doxo, 1 μm) (right) (600×, scale bar: 50 μm). (I) Immunoblotting analysis of cytoplasmic (C) and nuclear (N) fraction in TD cells after 1 μm of doxorubicin treatment (Doxo), PARP and α‐tubulin for equal protein loading in nuclear fraction and cytoplasmic fraction respectively. Mean ± SD, Student’s *t*‐test, *P* < 0.05(*), *P* < 0.01(**), *P* < 0.001(***) and *P* < 0.0001(****), *n* = 3.

To examine whether YAP activity promotes *AXL* expression, we depleted YAP/TAZ in TD cells and determined *AXL* expression thereafter. As expected, the significant repression of *AXL* resulted from depletion of YAP or TAZ along with a marked suppression of connective tissue growth factor (*CTGF*), a well‐established direct downstream target of YAP/TAZ [40] (Fig. 3A). The dependency of *AXL* expression on YAP/TAZ upon doxorubicin treatment, was confirmed by lower AXL protein levels following the depletion of YAP by siRNA (Fig. 3B). In contrast, the ectopic expression of constitutively active mutant of YAP (YAP8SA: inhibitory phosphorylation dead mutant) [41] was sufficient to induce *AXL* and *SERPINE1* (Fig. 3C), which was further validated by immunoblotting analysis (Fig. 3D). Therefore, reactivating the Hippo signaling pathway to inhibit YAP/TAZ transcriptional activation with two independent chemical inhibitors (dasatinib, an SRC inhibitor [42] or Y27632, a ROCK inhibitor [43]) markedly suppressed *AXL* expression in A549 and TD cells (Fig. 3E). Suppression of YAP/TAZ action by knockdown or inhibitor treatment clearly repressed AXL expression upon doxorubicin treatment (Fig. 3F). Therefore, the upregulation of *AXL* induced by doxorubicin treatment or present in doxorubicin‐resistant cancer cells (Fig. 2) and YAP‐dependent *AXL* expression (Fig. 3) suggests that YAP/TAZ activation may occur by doxorubicin treatment alone. Moreover, as expected, the GTIIC reporter was significantly upregulated in TD cells (Fig. 3G), which was coupled with a clear nuclear translocation of YAP in TD cells after doxorubicin treatment (Fig. 3H,I).

### 3.4 AXL expression induces doxorubicin resistance in TD cells

Consistent with the data presented in Fig. 1, high YAP/TAZ activity derived from the inactivation of the Hippo signaling pathway is known to be linked to EMT [44, 45]. Based on the high YAP activity in TD cells (Fig. 1D,G), we examined whether two clear mesenchymal characteristics of TD cells – high invasiveness [8, 36] and chemoresistance [7, 10] – were affected by high YAP activity.

**Fig. 4:**
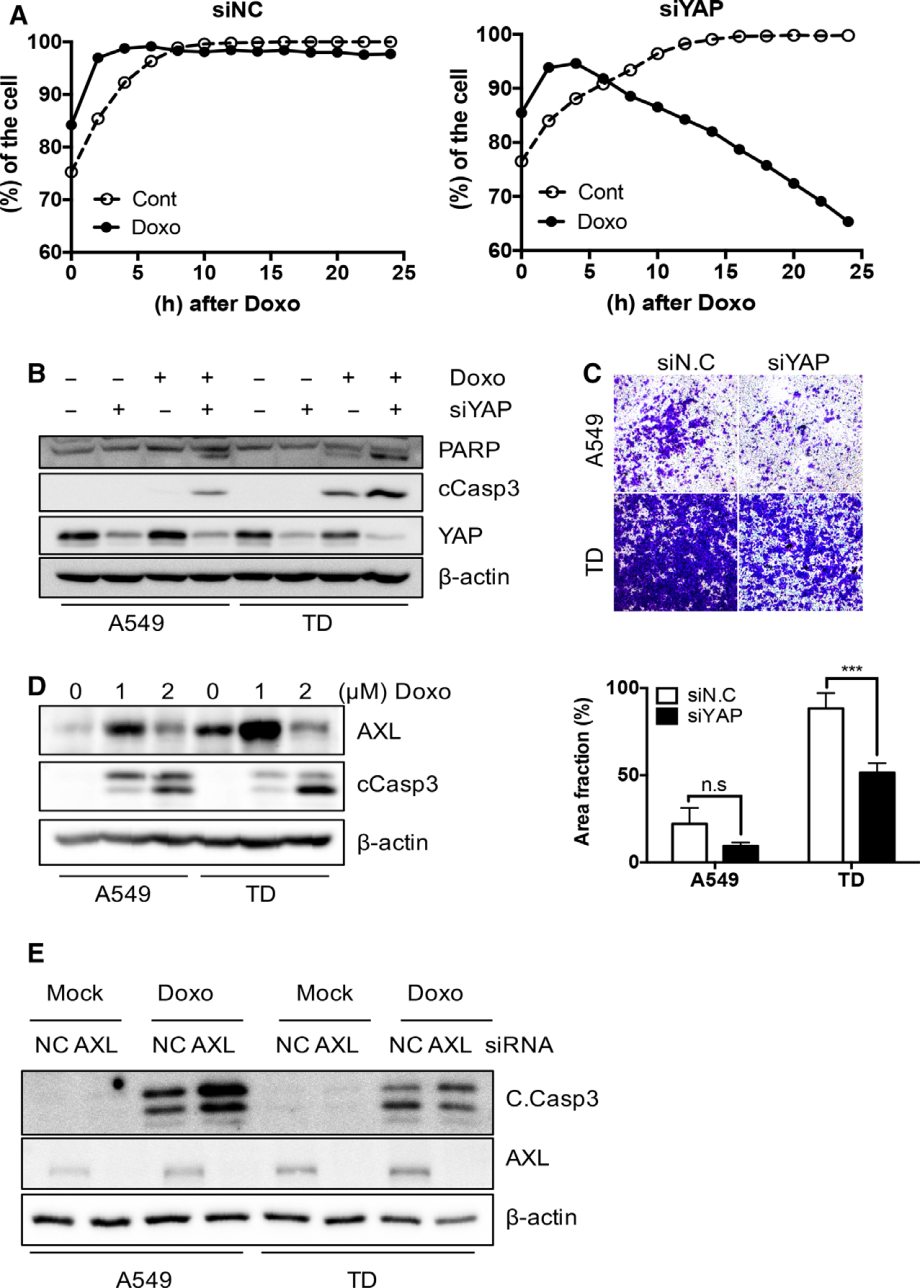
*AXL* expression for doxorubicin resistance in TD cells. (A) Cell growth of TD cells determined by JuLI stage, after introducing control (siNC: left) or YAP (siYAP: right) siRNA at indicative times after 2 μm doxorubicin treatment (Doxo). (B) Immunoblotting analysis for indicative proteins in A549 and TD cells after introduction control (–) or YAP (siYAP) siRNA, 24 h after 2 μm of doxorubicin (Doxo) treatment (C) Representative images of two chamber invasion assay of A549 and TD cells after introducing control (siNC) or YAP (siYAP) siRNA treatment (top) and graphical presentation of invaded area (bottom). (D) Immunoblotting of A549 and TD cells after doxorubicin (Doxo, 1 or 2 μm) treatment, β‐actin for equal protein loading, (E) Immunoblotting of the indicative proteins in A549 and TD cells of introducing control (siNC) or YAP (siYAP) siRNA treatment at 24 h after doxorubicin (Doxo, 2 μm) treatment, Mean ± SD, Student’s *t*‐test, *P* < 0.05(*), *P* < 0.01(**), *P* < 0.001(***) and *P* < 0.0001(****), n.s.: not significant, *n* = 3.

Chemoresistance to doxorubicin was first assessed based on cell growth potential. Although this potential remained unaffected even under doxorubicin treatment (siNC, Fig. 4A), significant attenuation of cell growth after depletion of YAP (siYAP) was observed after doxorubicin treatment (siYAP, Fig. 4A), which corresponded with high active caspase 3 activity due to YAP knockdown via doxorubicin treatment (Fig. 4B). Moreover, the depletion of YAP also inhibited cell invasiveness (Fig. 4C), suggesting that YAP‐dependent gene responses promoted chemoresistance and high invasiveness. *AXL* and *SERPINE1* were associated with downstream YAP signals via transcriptome analysis of doxorubicin‐resistant and mesenchymal‐type lung cancer cells (Fig. 2A–D); therefore, we investigated the effect of the knockdown of each gene on invasiveness or chemoresistance. Consistent with recent studies reporting that *AXL* upregulation contributed to target and conventional chemotherapy resistance [28, 29, 30], TD cells exhibited attenuated apoptotic responses coupled with *AXL* induction upon doxorubicin treatment compared with A549 cells and were sensitized to doxorubicin treatment by *AXL* depletion (Fig. 4D,E). In contrast, *AXL* knockdown had negligible effects on cell invasion (data not shown) and MMP2 and MMP9 activity (Fig. S3A,B). Additionally, knockdown of *SERPINE1* had an insignificant effect on chemoresistance (Fig. S3C,D) but did affect the invasiveness of TD cells (Fig. S3E). These results suggest that YAP‐dependent *AXL* expression contributes to doxorubicin resistance in doxorubicin‐treated TD cells.

### 3.5 TGFβ‐SMAD4 axis primes AXL expression upon doxorubicin treatment

**Fig. 5:**
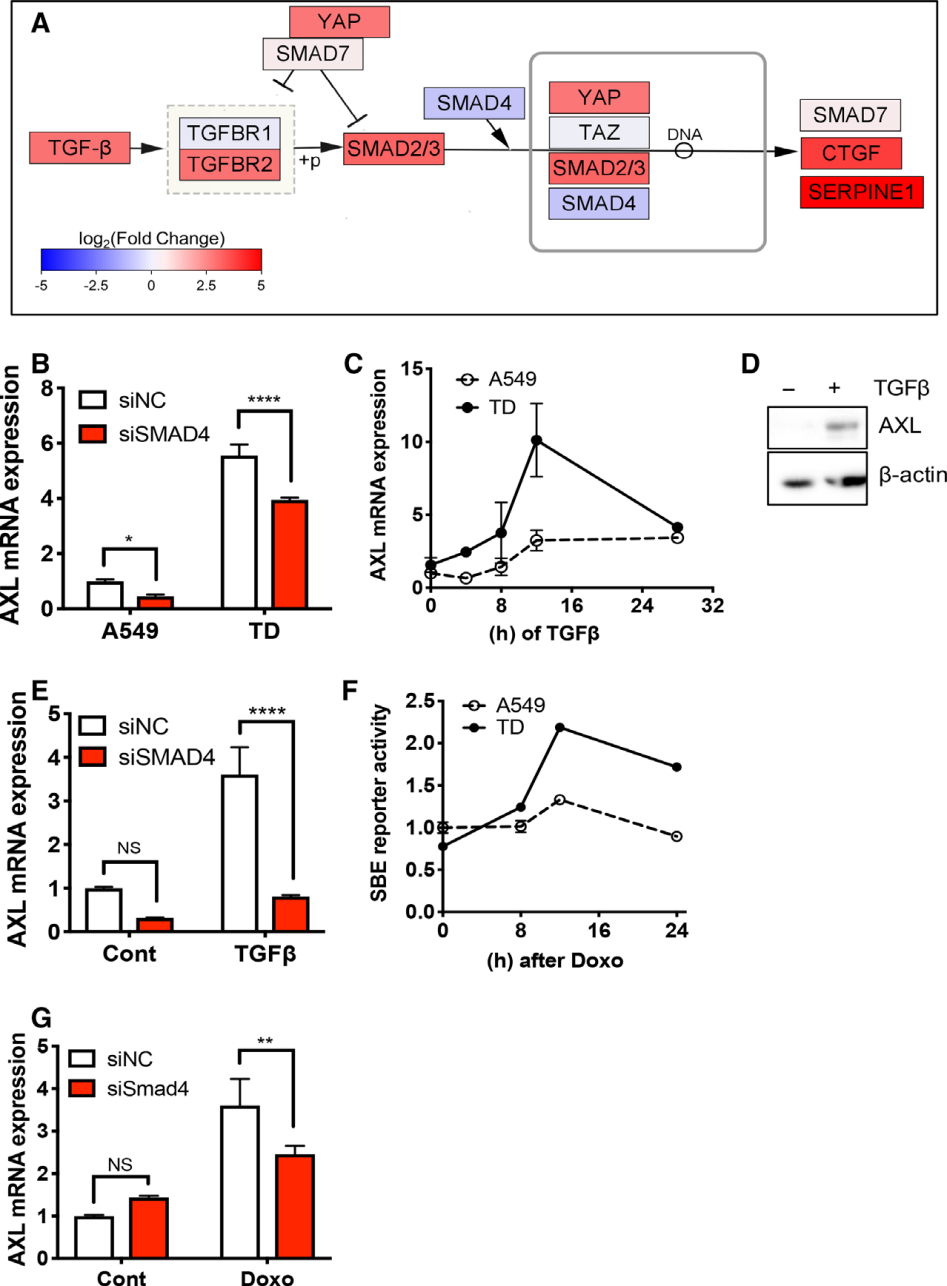
TGFβ‐SMAD4 axis to prime *AXL* expression upon doxorubicin. (A) Simplified TGFβ‐SMAD axis in Hippo signaling pathway. Each gene is colored according to log_2_ fold change values between high *AXL* and low *AXL* patient groups. (B,C) Real‐time PCR analysis of *AXL* in A549 and TD cells after introducing control (siNC) or SMAD4 (siSMAD4) siRNA (B) or at indicative time after 5 ng·mL^−1^ of TGFβ treatment (D) Immunoblotting analysis for *AXL* protein in TD cell upon TGFβ treatment (5 ng·mL^−1^). (E) Real‐time PCR analysis of *AXL* in A549 and TD cells, introducing control (siNC) or SMAD4 24 h after 5 ng·mL^−1^ of TGFβ treatment. (F) Reporter assay of SBE promoter activity at the indicative time after 1 μm of doxorubicin treatment (Doxo). (G) Real‐time PCR analysis of *AXL* in TD cells, introducing control (siNC) *AXL* or SMAD4 (siSMAD4) siRNA after doxorubicin treatment (Doxo, 1 μm). Mean ± SD, Student’s *t*‐test, *P* < 0.05(*), *P* < 0.01(**), *P* < 0.001(***) and *P* < 0.0001(****), NS: not significant, *n* = 3.

Upon doxorubicin treatment, *AXL* expression (Fig. 2G) and nuclear YAP levels (Fig. 3H) were significantly elevated in TD cells relative to A549 cells. This suggested that the transcriptional dysregulation of upstream signaling, which was accompanied by EMT, contributed to YAP/TAZ activity and subsequent *AXL* induction. Therefore, we searched for other factors associated with the Hippo signaling pathway that could affect YAP‐dependent gene responses in lung cancer cells with high *AXL* expression. Lung cancer cell lines were divided into high‐, intermediate‐ and low‐*AXL* groups based on the tertiles of the basal *AXL* expression level (Fig. S4A) and we visualized the differential expression between the high‐ and low‐*AXL* groups in the KEGG Hippo signaling pathway (Fig. 5A). Interestingly, TGFβ and SMAD2/3 were significantly upregulated in the high‐*AXL* group (Fig. 5A), suggesting that the activation of the TGFβ‐SMAD signaling pathway might contribute to YAP‐dependent *AXL* induction. To test the relevance of TGFβ‐SMAD signaling on *AXL* expression, we depleted SMAD4 to abolish TGFβ‐dependent gene expression and monitored *AXL* expression in our cell model. As shown in Fig. 5B, *AXL* expression was moderately modulated by SMAD4 knockdown in A549 and TD cells (Fig. 5B and Fig. S4B). Surprisingly, TGFβ treatment markedly induced *AXL* expression in TD cells, whereas the effect of TGFβ on AXL expression was negligible in A549 cells (Fig. 5C,D). This pattern was similar in *ZEB1*, a typical TGFβ‐mediated gene (Fig. S4C). The effect of TGFβ signaling on *AXL* expression was fully abrogated by SMAD4 knockdown, suggesting that the TGFβ‐SMAD axis contributes to *AXL* expression (Fig. 5E). Based on these results, we hypothesized that increased TGFβ‐SMAD signaling in mesenchymal‐type lung cancer cells after doxorubicin treatment potentiates YAP‐dependent *AXL* expression. As predicted, doxorubicin treatment increased SMAD binding element (SBE) reporter activity in TD cells in a time‐dependent manner (Fig. 5F). Moreover, SMAD4 depletion partially attenuated *AXL* or *SERPINE1* induction upon doxorubicin treatment (Fig. 5G and Fig. S4D).

### 3.6 TGFβ‐SMAD4 primes YAP‐dependent AXL expression

**Fig. 6:**
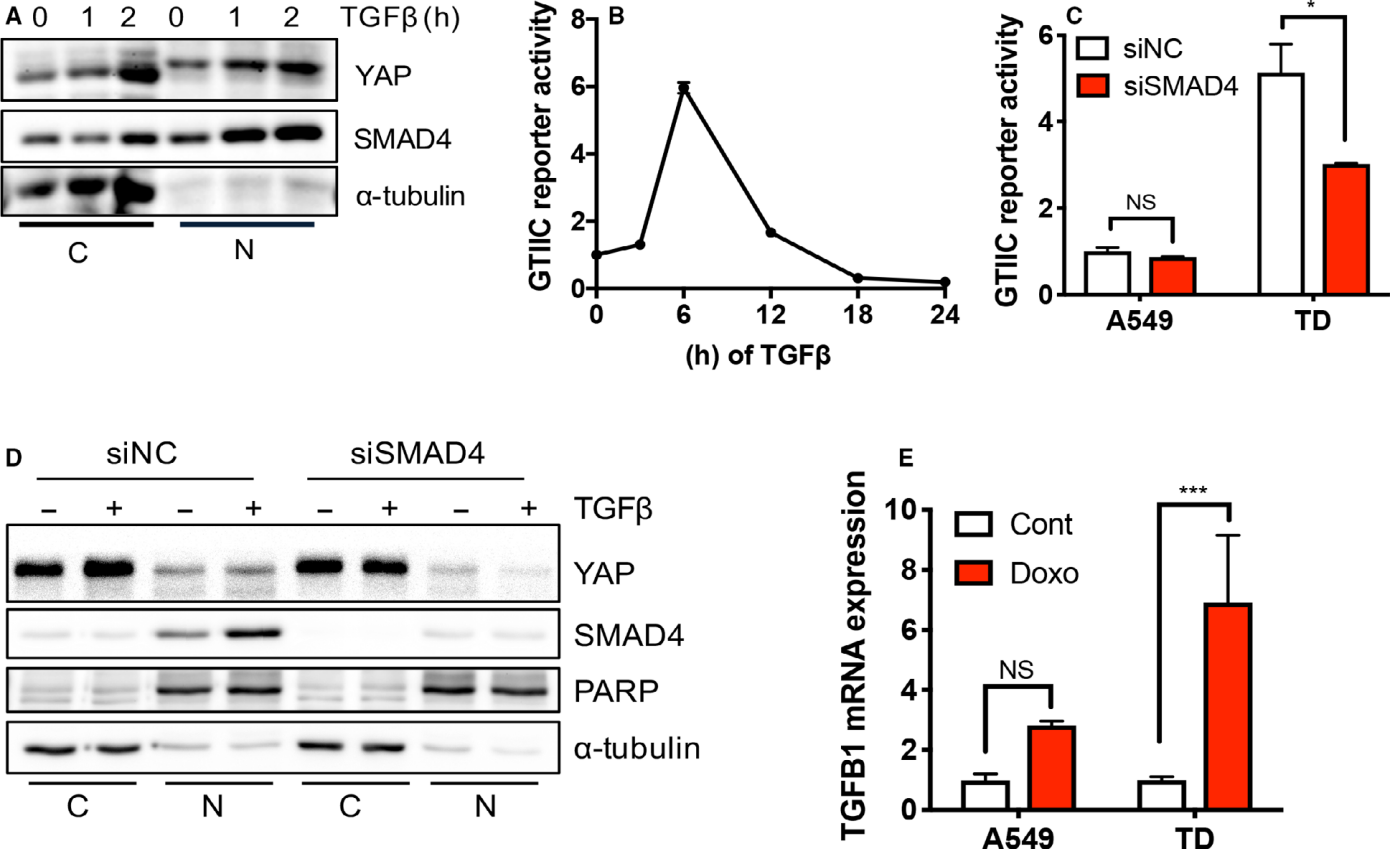
TGFβ‐SMAD4 primes YAP‐dependent *AXL* expression. (A) Immunoblotting analysis of cytoplasmic (C) and nuclear (N) fraction in TD cells at indicative time after 5 nm of TGFβ treatment, α‐tubulin for equal protein loading. (B,C) Reporter assay of GTIIC promoter activity in TD cell at indicative time after 5 ng·mL^−1^ TGFβ treatment (B) and in A549 and TD cells after introducing control (siNC) or SMAD4 (siSMAD4) siRNA. (D) Immunoblotting analysis of cytoplasmic (C) and nuclear (N) fraction in TD cells with siRNA of control (siNC) or SMAD4 (siSMAD4) 24 h after 5 nm TGFβ treatment, PARP and α‐tubulin for equal protein loading in nuclear fraction and cytoplasmic fraction respectively. (E) Real‐time PCR analysis of *TGFB1* in A549 and TD cells after doxorubicin treatment (Doxo, 1 μm), Mean ± SD, Student’s *t*‐test, *P* < 0.05(*), *P* < 0.01(**), *P* < 0.001(***) and *P* < 0.0001(****), NS, not significant, *n* = 3.

TGFβ is involved in the induction of EMT and stromal environment modulation, both of which are essential for tumor progression [46]. Moreover, cancer cells may be chronically exposed to TGFβ in the tumor microenvironment [37, 47]. Under these conditions, additional TGFβ stimulation further activated the TGFβ‐SMAD axis in mesenchymal cancer cells, resulting in increased YAP/TAZ activity. This was evidenced by the time‐dependent increase in YAP levels in the nucleus coupled with SMAD4 (Fig. 6A) and GTIIC reporter activity (Fig. 6B) upon TGFβ treatment. Even basal levels of YAP reporter activity were significantly modulated by SMAD4 knockdown in TD cells (Fig. 6C), suggesting that constant SMAD4‐dependent gene transcription contributed to maintaining high YAP activity in mesenchymal‐type lung cancer cells. Given that YAP/TAZ forms a protein complex with SMAD2/3 and SMAD4 and promotes nuclear translocation [48], the interaction between TGFβ‐SMAD4 and YAP/TAZ has been extensively characterized [49, 50]. Interestingly, a wild type (YAPWT) and a constitutively active YAP mutant (YAP8SA) failed to affect SBE reporter activity (Fig. S5), suggesting that the TGFβ‐SMAD axis might uni‐directionally prime YAP/TAZ‐dependent gene expression.

### 3.7 Clinical significance of AXL in mesenchymal‐type lung cancer patients

**Fig. 7:**
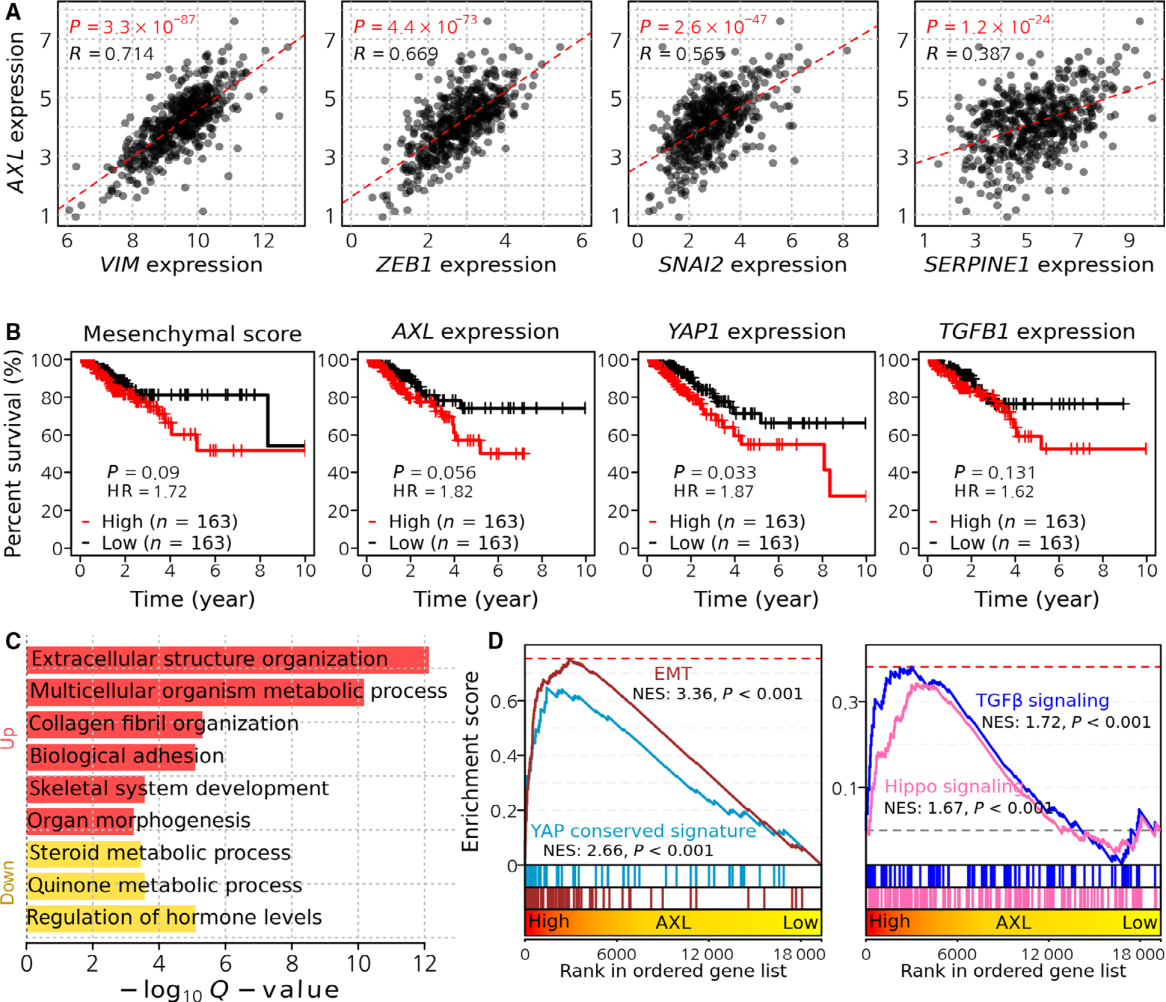
Clinical significance of *AXL* in the lung cancer patients. (A) Correlation between *AXL* expression and *VIM*, *ZEB1*, *SNAI2* and *SERPINE1* expression levels in TCGA lung adenocarcinoma patients. (B) Kaplan–Meier curves showing recurrence‐free survival time of lung adenocarcinoma patients. Patients were divided into three groups (high, intermediate, low) by mesenchymal score, expression levels of *AXL*, *YAP1* and *TGFB1*. High and low groups were used for comparison. (C) Significantly enriched functions in upregulated DEG in high *AXL* patients. Hypergeometric tests were performed using gene annotation in the biological process of the Gene Ontology database, yielding enrichment scores, defined as −log10 (*q*‐value). (D) GSEA plots showing the enrichment of EMT and YAP signatures (left) and TGFβ and Hippo signaling pathway (right) in a ranked list of genes differentially expressed between high and low AXL groups in TCGA lung adenocarcinoma patients.

As determined by our isogenic cell‐line model, AXL expression in mesenchymal‐type lung cancer cells, which confers doxorubicin resistance, was upregulated by YAP/TAZ activity primed by the TGFβ‐SMAD axis. These results were evaluated in primary tumors using the transcriptome data of 515 lung cancer patients from TCGA database. Similar to the high expression of *AXL* in TD cells and its close correlation to YAP/TAZ activity (Figs 2 and 3), *AXL* expression was closely correlated to typical EMT genes including *VIM* (encoding vimentin), *SNAI2* (encoding SLUG), *ZEB1, ZEB2*, *SERPINE1* and *CTGF* (Fig. 7A and Fig. S6A). Given that our results suggested that the upregulation of *AXL* conferred therapy‐resistant mesenchymal properties, we investigated the association between *AXL* expression and recurrence‐free patient survival. We found that a higher expression of *AXL* and higher mesenchymal scores were strongly associated with a poor prognosis, similar to other typical EMT genes and *YAP1* (Fig. 7B and Fig. S6B). To determine further whether *AXL* expression was indicative of mesenchymal properties in primary tumors, the patients were divided into high‐, intermediate‐ and low‐*AXL* groups based on the tertiles of *AXL* expression (Fig. S6C), after which the differentially expressed genes (DEG) between groups were examined (Fig. 5A). Functional enrichment analysis indicated that the organizing functions of cell structure, which are mostly affected during EMT [51], were significantly upregulated in high‐*AXL* patients (Fig. 7C). As expected, EMT and YAP signatures were strongly enriched in high‐*AXL* patients, thereby confirming the YAP‐dependent expression of *AXL* in mesenchymal‐type lung cancer cells (Fig. 7D). Consistent with the priming effect of TGFβ‐SMAD for YAP‐dependent *AXL* expression (Figs 5 and 6), the TGFβ signaling and Hippo signaling were also highly enriched in high‐*AXL* patients (Fig. 7D).

## 4 Discussion

Most targets responsible for acquired chemoresistance in cancers identified through extensive molecular mechanistic studies are poorly druggable, making it particularly challenging to identify therapeutic agents for chemosensitization [4]. The constant activation of YAP occurs in parallel with EMT to promote survival gene responses and has therefore been suggested to be an important molecular target. However, inhibiting protein binding to TEAD (i.e. its partner transcription factor) using small molecules remains particularly challenging despite many attempts. Therefore, the identification of *AXL* as a key survival factor in chemoresistant mesenchymal cancer and downstream YAP signaling factor is important because *AXL* (i.e. a receptor tyrosine kinase) is readily druggable via kinase inhibitors. In fact, many *AXL* inhibitors are currently being developed or investigated in clinical trials [22]. In this study, we used a doxorubicin‐resistant isogenic pair of lung cancer cell lines to demonstrate that YAP activation in mesenchymal‐type lung cancer cells (Fig. 1) induced *AXL* expression upon doxorubicin treatment (Fig. 2) and contributed to doxorubicin resistance (Figs 4 and 5). *AXL* induction in mesenchymal‐type lung cancer cells upon doxorubicin treatment (Fig. 2) was induced by the elevated nuclear translocation of YAP and subsequent increase in YAP transactivation (Fig. 3). Particularly, the activation of TGFβ signaling through SMAD4 after doxorubicin treatment (Fig. 5) contributed to YAP‐dependent *AXL* expression in mesenchymal‐type lung cancer cells (Fig. 6). These results suggest that the crosstalk between TGFβ (which is highly enriched in cancer microenvironments) and the YAP pathway synergizes with *AXL* expression during EMT to promote chemoresistance. Furthermore, TGFβ expression was promoted in mesenchymal‐type lung cancer cells (Fig. 6E), further enhancing this synergistic effect.

The crosstalk between TGFβ and YAP/TAZ has been extensively studied. YAP/TAZ regulates TGFβ signaling [52] and promotes SMAD2/3 nuclear translocation [48]. However, the ectopic expression of YAP (WT) and constitutively active YAP (YAP8SA) failed to affect SMAD4‐dependent gene responses (Fig. S6). Furthermore, TGFβ stimuli promoted the nuclear translocation of YAP (Fig. 6A) and YAP‐dependent gene responses, including *AXL* (Figs 5 and 6), suggesting that a TGFβ‐rich microenvironment favors YAP‐dependent *AXL* expression, which leads to chemoresistance. These results are consistent with a previous study that reported the positive effects of TGFβ on YAP transactivation [53]. It is also worth noting that the direct interaction of ZEB1 with the YAP/TEAD complex alters the YAP‐dependent gene expression profile, which favors therapy resistance and increases metastatic risk [54]. Additionally, the presence of SMAD2/3 in the YAP–TEAD complex coordinates transcriptional regulation, which promotes an aggressive metastasis response [55]. Similarly, we also observed that the loss of SMAD4 led to alterations in YAP‐dependent gene responses (Fig. S6B). Unfortunately, we did not confirm the presence of SMAD in the *AXL* promoters (data not shown). Therefore, it is still unclear whether TGFβ‐mediated SMAD elevate YAP‐dependent *AXL* expression directly or indirectly. Alternatively, microRNA (miR)‐34a, which downregulates *AXL* [56], was reportedly suppressed by TGFβ [57]. Thus, it would be interesting to quantify miR‐34a levels upon doxorubicin treatment to characterize the role of miR‐34a on *AXL* expression in mesenchymal‐type lung cancer cells compared with epithelial cancer cells.

## 5 Conclusions

Our results indicate that a doxorubicin‐induced increase in TGFβ expression contributes to YAP‐dependent *AXL* induction in mesenchymal‐type lung cancer cells, suggesting that alterations in EMT lead to chemoresistance by enhancing YAP‐dependent survival gene responses. Therefore, the modulation of TGFβ signaling is a promising chemosensitizing alternative strategy. Upregulation of the TGFβ/SMAD pathway primes YAP‐dependent *AXL* expression upon doxorubicin treatment to promote chemoresistance in mesenchymal‐type lung cancer cells.

## Conflict of interest

The authors declare no conflict of interest.

## Author contributions

HJ.C conceived the overall study design and led the experiments. JY.C and H.L conducted the experiments and database analyses, and provided critical discussion of the results. OS.K and HJ.K provided the initial preliminary data. All authors contributed to manuscript writing and revision and endorsed the final manuscript.

### Peer Review

The peer review history for this article is available at https://publons.com/publon/10.1002/1878‐0261.12857.

## Supporting information


**Fig. S1.** (A) CCLE lung cancer cell lines ranked by mesenchymal score. Distribution of mesenchymal score is shown in the right panel. Cell lines were divided into tertiles by mesenchymal score (mesenchymal, intermediate, epithelial groups). (B) Distribution of mesenchymal score of 78 lung cancer cell lines in GSE4824. Cell lines were divided into three groups according to mesenchymal score (mesenchymal, intermediate, epithelial groups). (C) ZEB2 mRNA expression level in A549, TD, H358 and H1299 cells. (D) Immunostaining of YAP in A549 and TD cells.
**Fig. S2.** (A) Association between cell‐line enrichment scores of oncogenic signatures and drug sensitivity (AUC) to doxorubicin, topotecan and gemcitabine in 181 lung cancer cell lines in the CTD^2^ database. (B) Lung cancer cell lines ranked by doxorubicin sensitivity. Distribution of area under the curve (AUC) is shown in the right panel. Cell lines were divided into tertiles of AUC (doxorubicin‐resistant, ‐intermediate and ‐sensitive groups). (C) Correlation between cell‐line sensitivity (AUC) to doxorubicin and enrichment score of YAP signature (left) or mesenchymal signature (right) in lung cancer cell lines. (D) SERPINE1 messenger (m)RNA expression upon indicative concentration of doxorubicin (Doxo) treatment. (E) AXL (left) and SERPINE1 (right) mRNA expression upon indicative concentration of etoposide (Eto) treatment.
**Fig. S3.** (A) Representative images of zymography assay of A549 and TD cells after AXL siRNA treatment. (B) AXL mRNA expression in A549 and TD cells. (C,D) Immunoblotting analysis for PARP‐1 in TD cells, introducing of control (siNC) or SERINE1 (siSERPINE1) after treatment of doxorubicin (C: Doxo, 2 μm) or, etoposide (D: Eto, 40 μm). (E) Representative images of two‐chamber invasion assay of A549 and TD cells with control (siNC) or SERINE1 (siSERPINE1) (left) and graphical presentation of invaded area (% of Area) (right).
**Fig. S4.** (A) Distribution of *AXL* expression levels of 181 lung cancer cell lines in CCLE data. Cell lines were divided into tertiles of *AXL* expression (high, intermediate and low AXL groups). (B) Realtime PCR analysis for SMAD4 mRNA expression in A549 and TD cells with control (siNC) or SMAD4 (siSMAD4) siRNA. (C) Real‐time PCR analysis for ZEB1 mRNA expression in A549 and TD cells after indicative time of TGFβ treatment (5 ng/mL). (D) Real‐time PCR analysis for SERPINE1 mRNA expression with control (siNC) or SMAD4 (siSMAD4) siRNA, NS, not significant.
**Fig. S5.** Graphical presentation of Reporter activity of GTIIC and SBE with wild type (YAP WT) or constitutively active mutant (YAP8SA) of YAP.
**Fig. S6.** (A) Correlation between *AXL* expression and *TGFB1*, *ZEB2*, *CTGF* and *YAP1* expression levels in 515 lung adenocarcinoma patients in TCGA data. (B) The Kaplan–Meier curves showing recurrence‐free survival time of lung adenocarcinoma patients. Patients were divided into tertiles (high, intermediate, low) by the expression levels of *VIM*, *ZEB1*, *SNAI2* and SERPINE1. High and low groups were used for comparison. (C) Distribution of *AXL* expression levels of lung adenocarcinoma patients in TCGA data. Patients were divided into high, intermediate, and low *AXL* groups based on the basal expression level of *AXL*.
**Fig. S7.** Uncut immunoblotting data used in these studies.

**Table S1.** Correlation coefficient between cell‐line mesenchymal score (Taube et al., Grojer et al. and Byers et al.) and cell‐line oncogenic score calculated by using ssGSEA with 189 oncogenic signature gene sets.
**Table S2.** Correlation coefficient between cell‐line response (AUC) to doxorubicin and cell‐line oncogenic score.

## Data Availability

The datasets used and/or analyzed herein can be obtained from the corresponding author upon request.
