# Supplementary material for: TGFβ promotes YAP‐dependent AXL induction in mesenchymal‐type lung cancer cells
Source: Mol Oncol. 2020 Dec 5;15(2):679–96. doi: 10.1002/1878-0261.12857 (PMC7858114; doi:10.1002/1878-0261.12857)

Supplementary Figure legends

Figure S1 A. CCLE lung cancer cell lines ranked by mesenchymal score. Distribution of mesenchymal score is shown in the right panel. Cell lines were divided into tertiles by mesenchymal score (mesenchymal, intermediate, epithelial groups) **B.** Distribution of mesenchymal score of 78 lung cancer cell lines in GSE4824. Cell lines were divided into three groups according to mesenchymal score (mesenchymal, intermediate, epithelial groups) **C.** ZEB2 mRNA expression level in A549, TD, H358, and H1299 cells **D.** Immunostaining of YAP in A549 and TD cells

Figure S2 A. Association between cell-line enrichment scores of oncogenic signatures and drug sensitivity (AUC) to doxorubicin, topotecan, and gemcitabine in 181 lung cancer cell lines in the CTD^2 database. **B**. Lung cancer cell lines ranked by doxorubicin sensitivity. Distribution of AUC is shown in the right panel. Cell lines were divided into tertiles of AUC (doxorubicin-resistant, -intermediate, and -sensitive groups) **C**. Correlation between cell-line sensitivity (AUC) to doxorubicin and enrichment score of YAP signature (left) or mesenchymal signature (right) in lung cancer cell lines **D.** SERPINE1 mRNA expression upon indicative concentration of doxorubicin (Doxo) treatment **E.** AXL (left) and SERPINE1 (right) mRNA expression upon indicative concentration of etoposide (Eto) treatment

Figure S3 A. Representative images of zymography assay of A549 and TD cells after AXL siRNA treatment **B**. AXL mRNA expression in A549 and TD cells, **C and D.** Immunoblotting analysis for PARP-1 in TD cells, introducing of control (siNC) or SERINE1 (siSERPINE1) after treatment of doxorubicin (C: Doxo, 2μM) or , etoposide (D: Eto, 40μM) **E.** Representative images of two chamber invasion assay of A549 and TD cells with control (siNC) or SERINE1 (siSERPINE1) (left) and graphical presentation of invaded area (% of Area) (right)

Figure S4 A. Distribution of *AXL* expression levels of 181 lung cancer cell lines in CCLE data. Cell lines were divided into tertiles of *AXL* expression (AXL-high, -intermediate, and -low groups) **B.** Realtime PCR analysis for SMAD4 mRNA expression in A549 and TD cells with control (siNC) or SMAD4 (siSMAD4) siRNA **C.** Realtime PCR analysis for ZEB1 mRNA expression in A549 and TD cells after indicative time of TGFβ treatment (5ng/ml) **D.** Realtime PCR analysis for SERPINE1 mRNA expression with control (siNC) or SMAD4 (siSMAD4) siRNA, NS: Not significant

Figure S5 Graphical presentation of Reporter activity of GTIIC and SBE with wild type (YAP WT) or constitutively active mutant (YAP8SA) of YAP

Figure S6 A. Correlation between *AXL* expression and *TGFB1*, *ZEB2*, *CTGF*, and *YAP1* expression levels in 515 lung adenocarcinoma patients in TCGA data **B.** The Kaplan–Meier curves showing recurrence-free survival time of lung adenocarcinoma patients. Patients were divided into tertiles (high, intermediate, low) by the expression levels of *VIM*, *ZEB1*, *SNAI2*, and SERPINE1. High- and low groups were taken for comparison **C.** Distribution of *AXL* expression levels of lung adenocarcinoma patients in TCGA data. Patients were divided into AXL-high, -intermediate, and -low groups by the basal expression level of *AXL*

Figure S7 Uncut immunoblotting data used in these studies

Figure S1

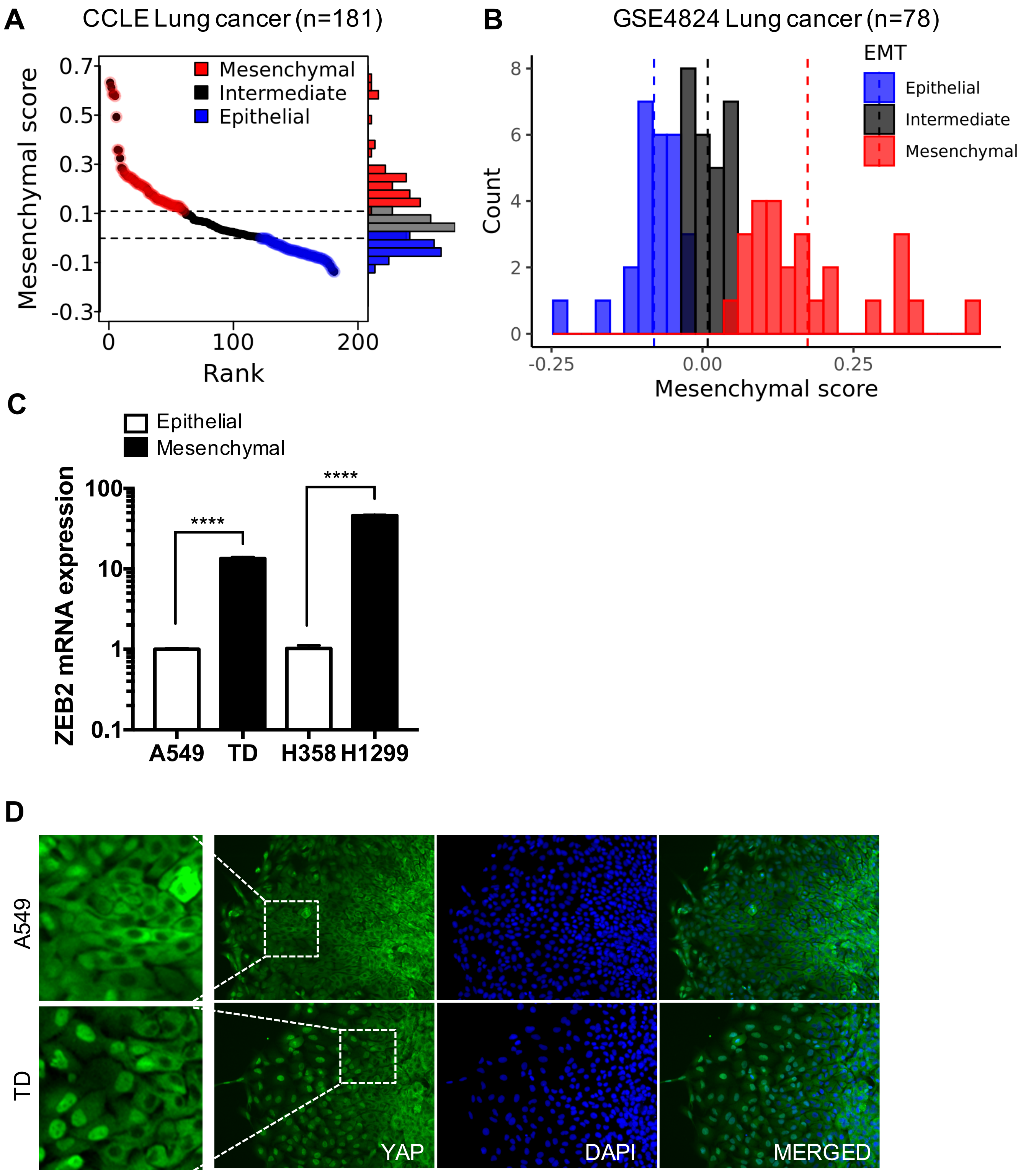
A
CCLE Lung cancer (n=181)
Mesenchymal
Intermediate
Epithelial
Mesenchymal score
0.7
0.5
0.3
0.1
-0.1
-0.3
0
100
200
Rank
B
GSE4824 Lung cancer (n=78)
EMT
Epithelial
Intermediate
Mesenchymal
Count
8
6
4
2
0
-0.25
0.00
0.25
Mesenchymal score
C
Epithelial
Mesenchymal
ZEB2 mRNA expression
100
10
1
0.1
****
****
A549
TD
H358
H1299
D
A549
TD
YAP
DAPI
MERGED

Figure S2

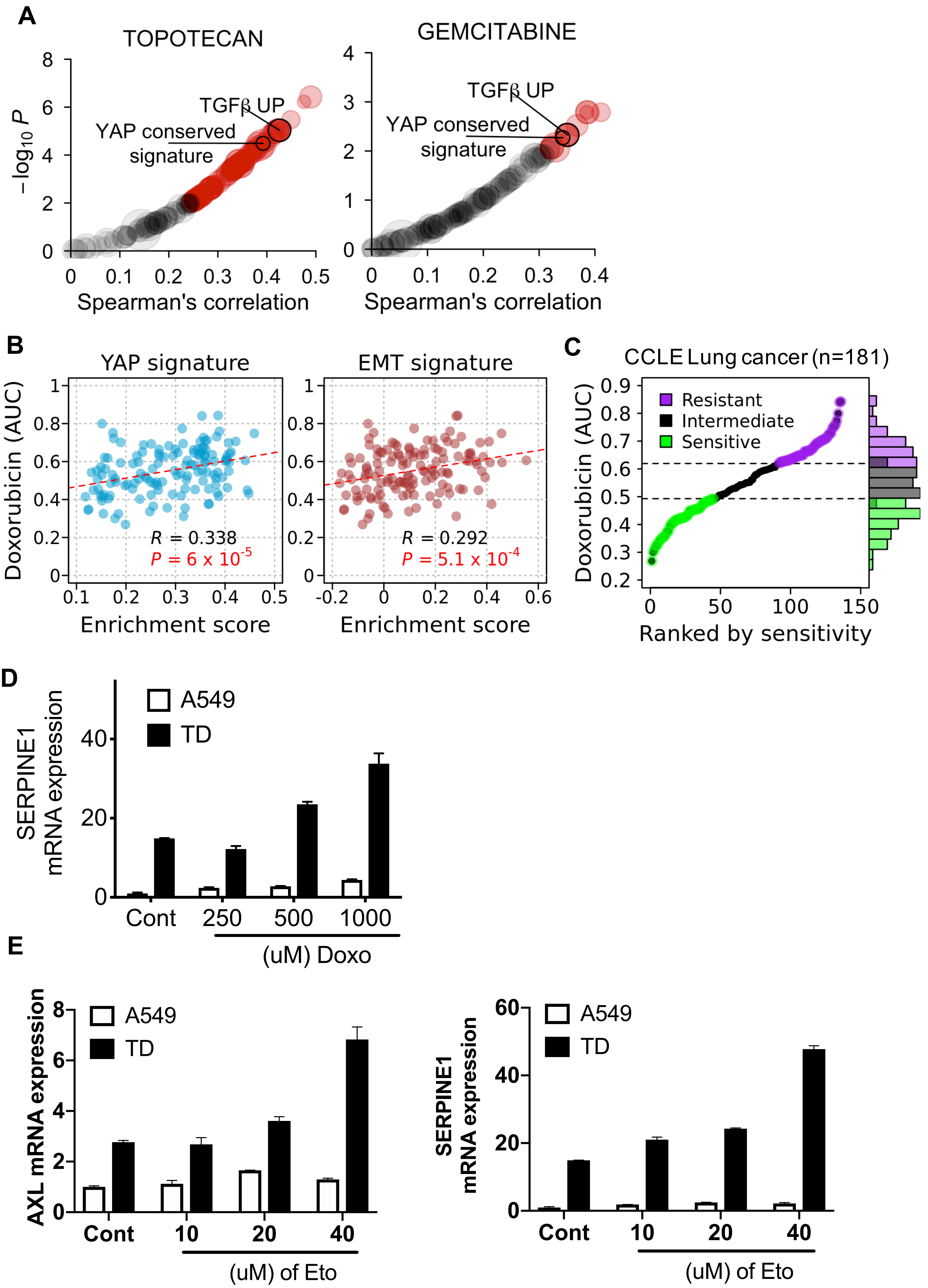

A
TOPOTECAN
GEMCITABINE
TGFβ UP
YAP conserved signature
−log10 P
Spearman's correlation
B
YAP signature
EMT signature
Doxorubicin (AUC)
Enrichment score
R = 0.338
P = 6 x 10-5
R = 0.292
P = 5.1 x 10-4
C
CCLE Lung cancer (n=181)
Resistant
Intermediate
Sensitive
Ranked by sensitivity
D
SERPINE1 mRNA expression
A549
TD
Cont
250
500
1000
(uM) Doxo
E
AXL mRNA expression
(uM) of Eto

Figure S3

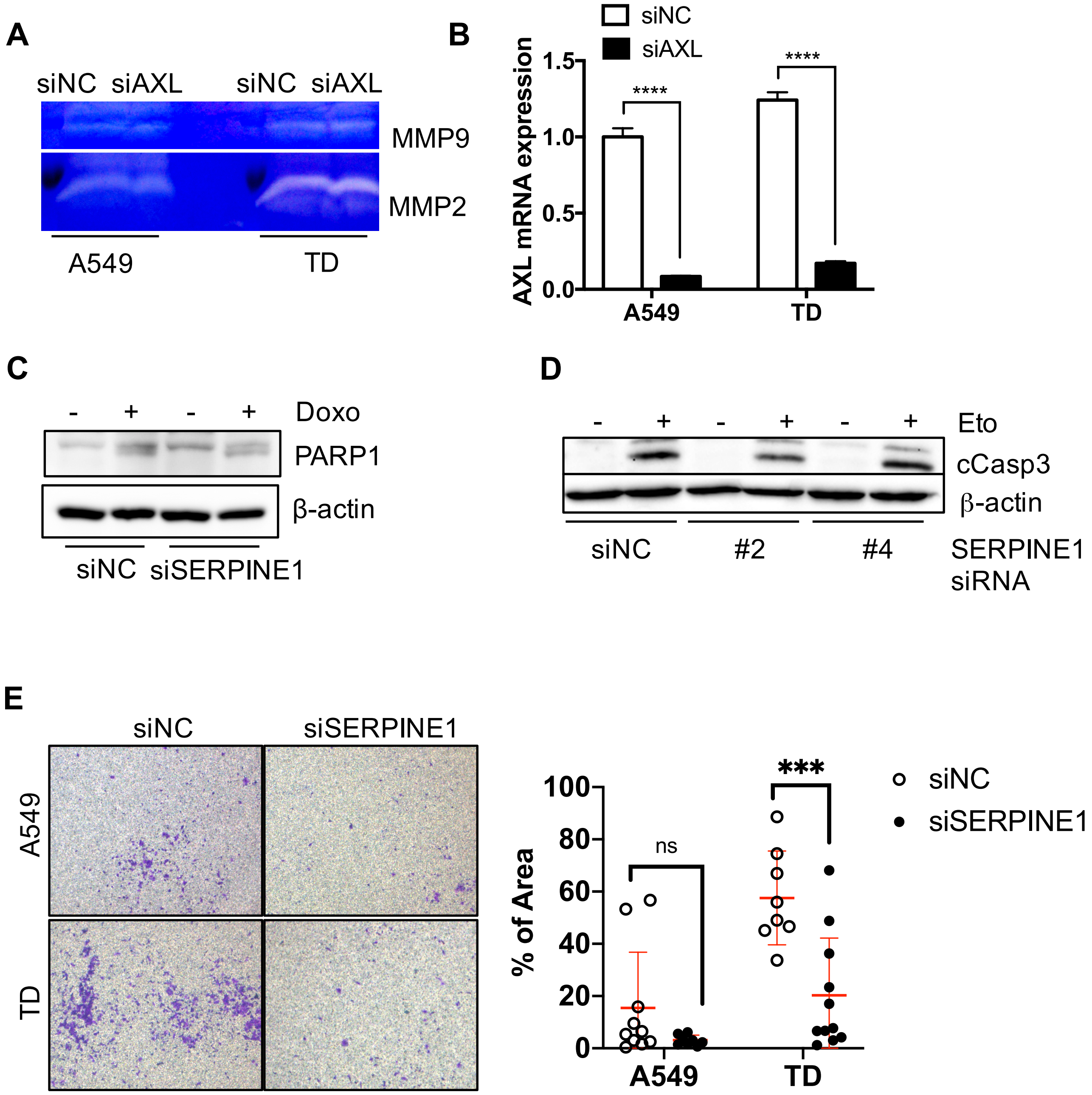
A
siNC siAXL siNC siAXL
MMP9
MMP2
A549
TD
B
siNC
siAXL
AXL mRNA expression
1.5
1.0
0.5
0.0
****
****
A549
TD
C
- + - + Doxo
PARP1
β-actin
siNC siSERPINE1
D
- + - + - + Eto
cCasp3
β-actin
siNC #2 #4 SERPINE1 siRNA
E
siNC siSERPINE1
A549
TD
% of Area
100
80
60
40
20
0
ns
***
A549
TD
siNC
siSERPINE1

Figure S4

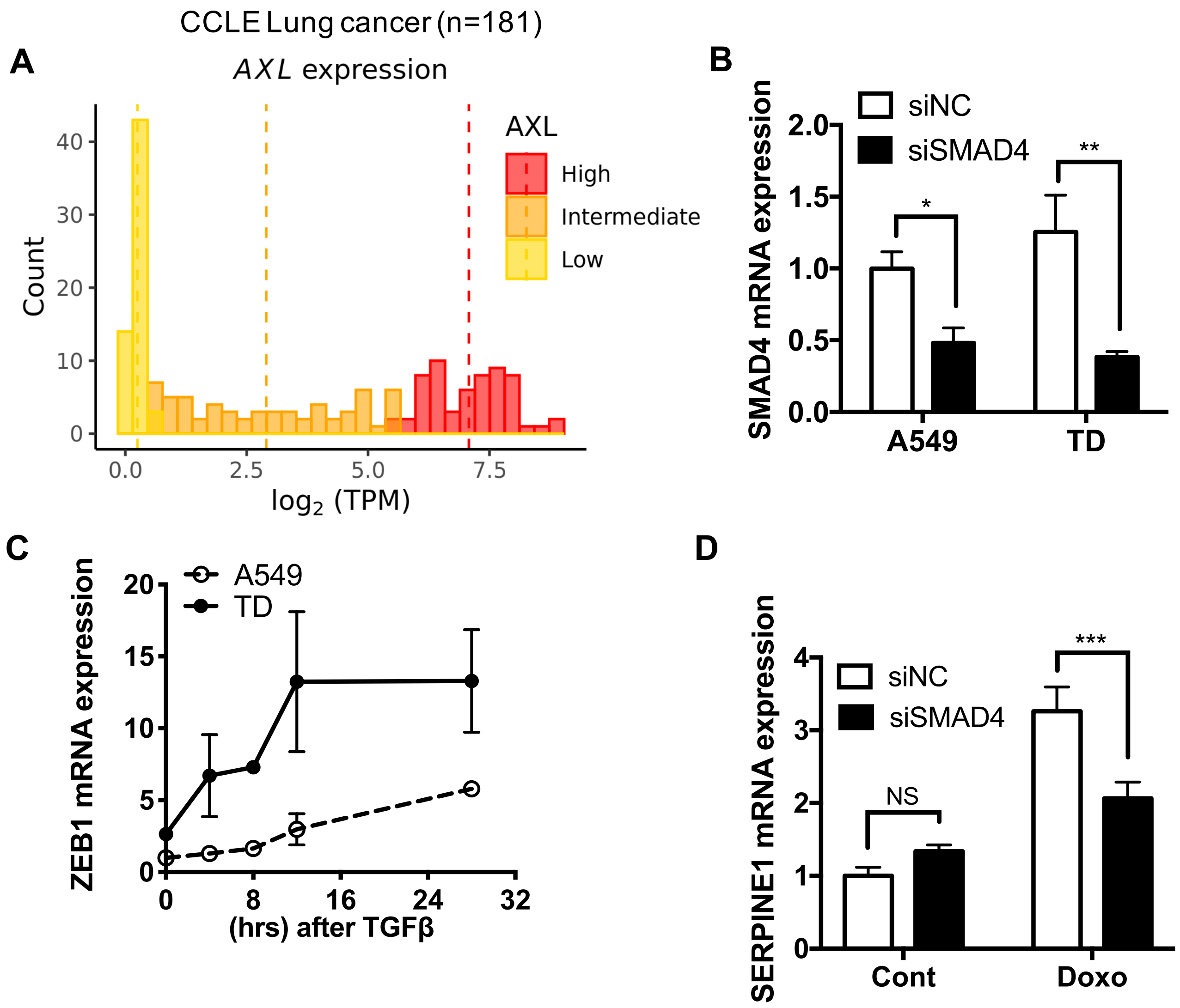
A
CCLE Lung cancer (n=181)
AXL expression
Count
log2 (TPM)
AXL
High
Intermediate
Low
0
10
20
30
40
0.0
2.5
5.0
7.5
B
SMAD4 mRNA expression
siNC
siSMAD4
0.0
0.5
1.0
1.5
2.0
*
**
A549
TD
C
ZEB1 mRNA expression
A549
TD
0
5
10
15
20
0
8
16
24
32
(hrs) after TGFβ
D
SERPINE1 mRNA expression
siNC
siSMAD4
0
1
2
3
4
NS
***
Cont
Doxo

Figure S5

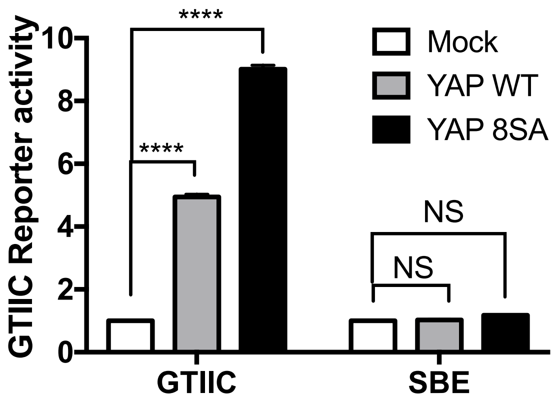

GTIIC Reporter activity
0
2
4
6
8
10
****
****
NS
NS
Mock
YAP WT
YAP 8SA
GTIIC
SBE

Figure S6

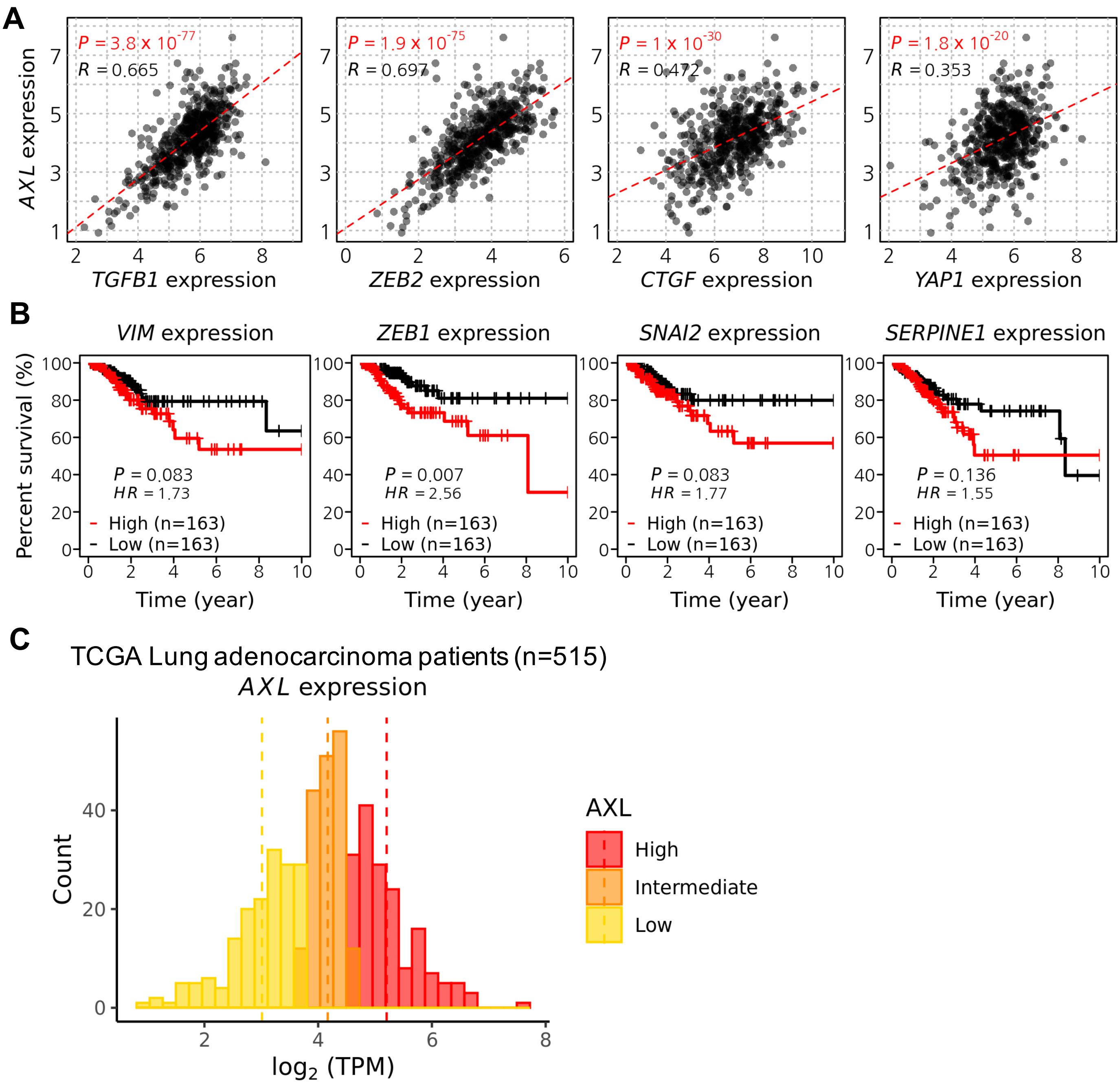
A
P = 3.8 x 10^-77
R = 0.665
P = 1.9 x 10^-75
R = 0.697
P = 1 x 10^-30
R = 0.472
P = 1.8 x 10^-20
R = 0.353
AXL expression
TGFB1 expression
ZEB2 expression
CTGF expression
YAP1 expression
B
VIM expression
ZEB1 expression
SNAI2 expression
SERPINE1 expression
Percent survival (%)
P = 0.083
HR = 1.73
P = 0.007
HR = 2.56
P = 0.083
HR = 1.77
P = 0.136
HR = 1.55
High (n=163)
Low (n=163)
Time (year)
C
TCGA Lung adenocarcinoma patients (n=515)
AXL expression
Count
log2 (TPM)
AXL
High
Intermediate
Low

Figure S7

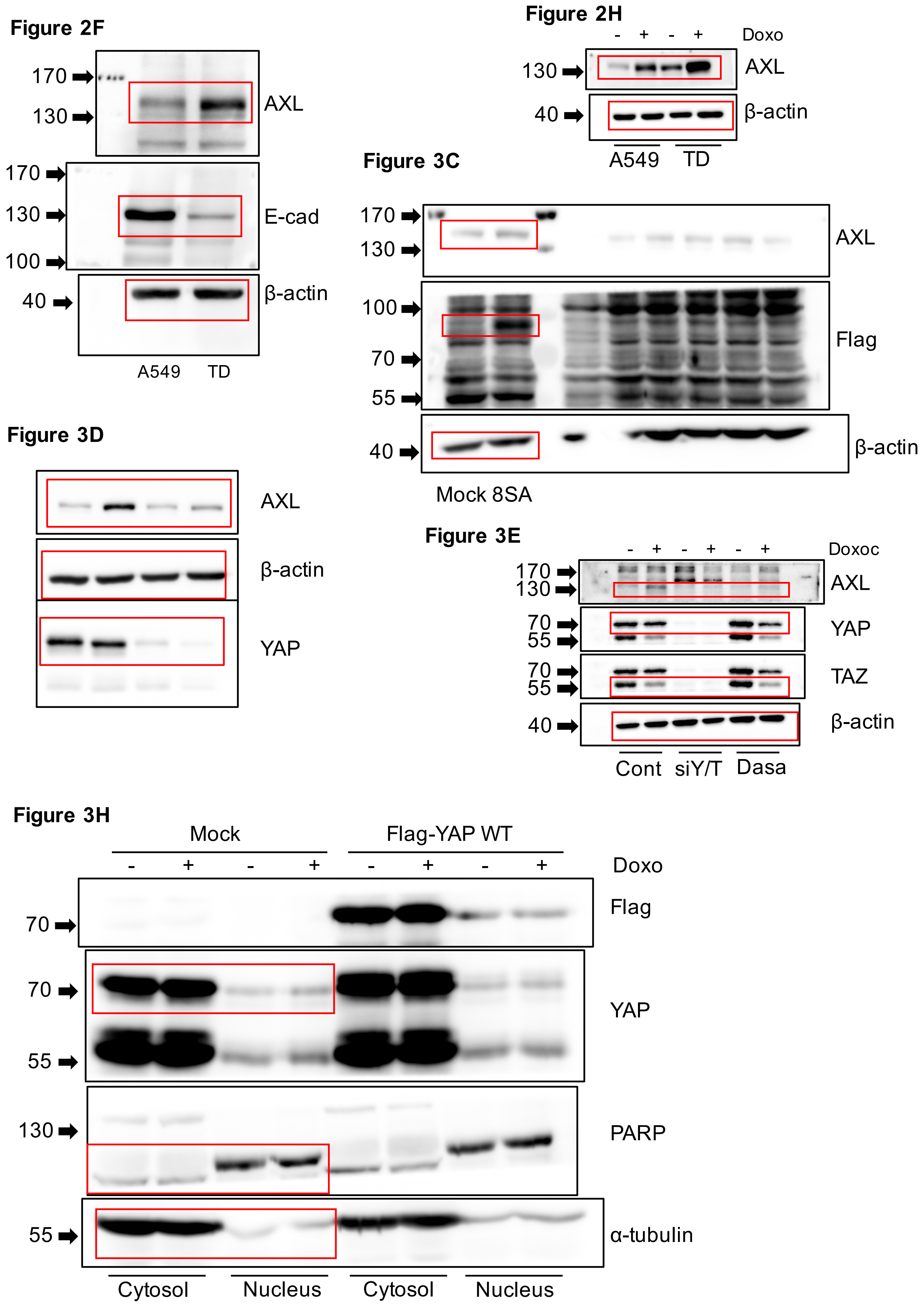
Figure 2F
170
130
AXL
170
130
100
E-cad
40
β-actin
A549
TD
Figure 2H
- + - +
Doxo
130
AXL
40
β-actin
A549
TD
Figure 3C
170
130
AXL
100
70
55
Flag
40
β-actin
Mock 8SA
Figure 3D
AXL
β-actin
YAP
Figure 3E
- + - + - +
Doxoc
170
130
AXL
70
55
YAP
70
55
TAZ
40
β-actin
Cont
siY/T
Dasa
Figure 3H
Mock
Flag-YAP WT
- + - + - + - +
Doxo
70
Flag
70
55
YAP
130
PARP
55
α-tubulin
Cytosol
Nucleus
Cytosol
Nucleus

Figure 4D

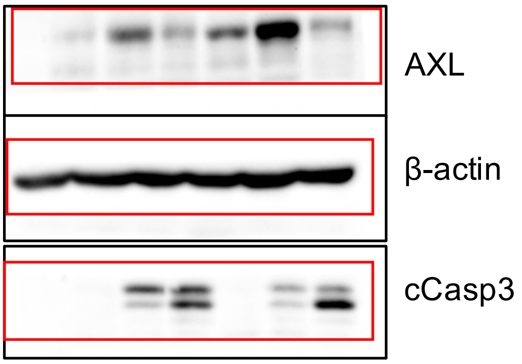

Figure 4E

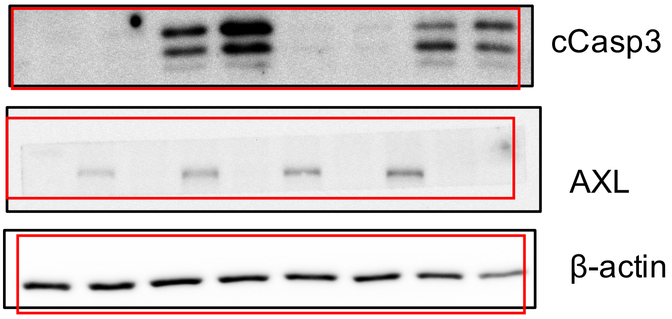

Figure 6A

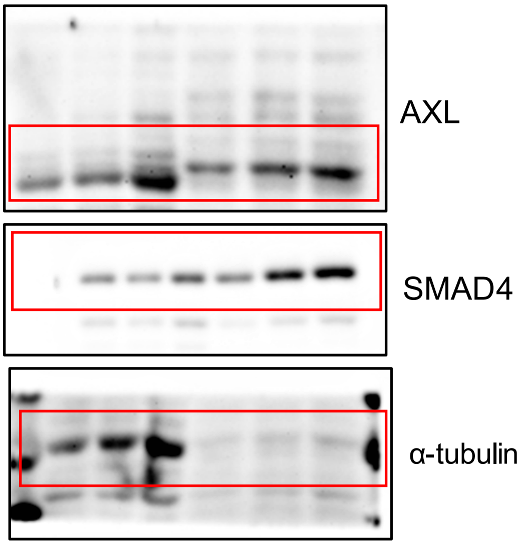

Supplement: Supplementary file 1 — Fig. S1. (A) CCLE lung cancer cell lines ranked by mesenchymal score. Distribution of mesenchymal score is shown in the right panel. Cell lines were divided into tertiles by mesenchymal score (mesenchymal, intermediate, epithelial groups). (B) Distribution of mesenchymal score of 78 lung cancer cell lines in GSE4824. Cell lines were divided into three groups according to mesenchymal score (mesenchymal, intermediate, epithelial groups). (C) ZEB2 mRNA expression level in A549, TD, H358 and H1299 cells. (D) Immunostaining of YAP in A549 and TD cells. Fig. S2. (A) Association between cell‐line enrichment scores of oncogenic signatures and drug sensitivity (AUC) to doxorubicin, topotecan and gemcitabine in 181 lung cancer cell lines in the CTD2 database. (B) Lung cancer cell lines ranked by doxorubicin sensitivity. Distribution of area under the curve (AUC) is shown in the right panel. Cell lines were divided into tertiles of AUC (doxorubicin‐resistant, ‐intermediate and ‐sensitive groups). (C) Correlation between cell‐line sensitivity (AUC) to doxorubicin and enrichment score of YAP signature (left) or mesenchymal signature (right) in lung cancer cell lines. (D) SERPINE1 messenger (m)RNA expression upon indicative concentration of doxorubicin (Doxo) treatment. (E) AXL (left) and SERPINE1 (right) mRNA expression upon indicative concentration of etoposide (Eto) treatment. Fig. S3. (A) Representative images of zymography assay of A549 and TD cells after AXL siRNA treatment. (B) AXL mRNA expression in A549 and TD cells. (C,D) Immunoblotting analysis for PARP‐1 in TD cells, introducing of control (siNC) or SERINE1 (siSERPINE1) after treatment of doxorubicin (C: Doxo, 2 μm) or, etoposide (D: Eto, 40 μm). (E) Representative images of two‐chamber invasion assay of A549 and TD cells with control (siNC) or SERINE1 (siSERPINE1) (left) and graphical presentation of invaded area (% of Area) (right). Fig. S4. (A) Distribution of AXL expression levels of 181 lung cancer cell lines in [file MOL2-15-679-s001.pdf]
